# Chemistry and biology of oxidatively damaged RNA nucleotides

**DOI:** 10.1039/d6cb00143b

**Published:** 2026-09-01

**Authors:** Kasturi Raorane, Marlies Weber, Virginie Marchand, Mark Helm, Yuri Motorin

**Affiliations:** a Université de Lorraine CNRS, IMoPA UMR7365 F-54000 Nancy France yuri.motorin@univ-lorraine.fr; b Johannes Gutenberg-University, Mainz Institute of Pharmacy and Biomedical Sciences Staudingerweg 555128 Mainz Germany mhelm@uni-mainz.de; c Université de Lorraine, SMP IBSLor Epitranscriptomics and RNA Sequencing Core Facility F-54000 Nancy France

## Abstract

Nucleic acid damage under oxidative stress conditions is a well-established phenomenon relevant to evolution of all living organisms. While well investigated for DNA, cellular RNAs are also a subject of extensive damage, featuring similar reactivity towards reactive oxygen species generated by external factors and cellular metabolism. The chemistry of DNA and RNA oxidative damage is rather complex, involving a variety of concomitant chemical reactions which are further exacerbated by secondary reactions that increase the already overwhelming list of chemically damaged nucleotides. The damage is of random character, typically resulting in sub-stoichiometrically damaged sites. This substantially complicates the analysis of RNA oxidation. Modern analytical techniques include deep sequencing-based protocols allowing precise mapping of such damaged residues in cellular RNAs. Increasing experimental evidence suggests vast and widespread biological consequences and highlights the importance of the metabolism of oxidized RNA in a variety of cellular processes. The main effects are expected at the level of translation, since all key players of the translational machinery, mRNA, rRNA and tRNAs are prominent oxidation targets. In this review we present current achievements in the analysis of RNA oxidation/damage chemistry, traditional and modern methods allowing RNA oxidation analysis and current view on the biological consequences of RNA oxidation in the living cell.

## Abstract 2

Review of current achievements in the analysis of RNA oxidation/damage chemistry, traditional and modern methods allowing RNA oxidation analysis and current view on the biological consequences of RNA oxidation in the living cell.

## Graphical abstract

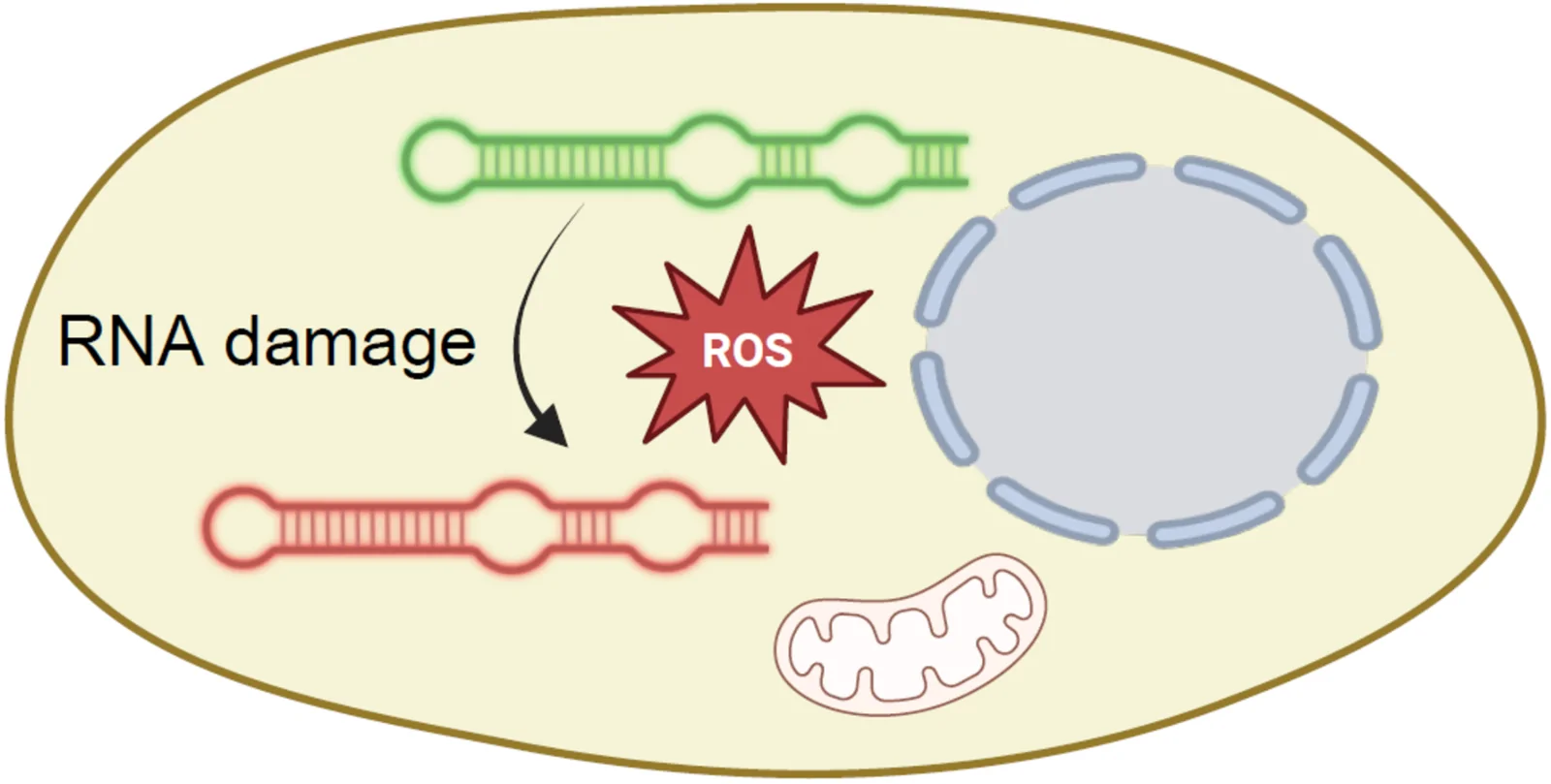

## Introduction

RNA damage through oxidation, though as frequent as oxidation of DNA and proteins, remained largely understudied in the past decades. With much attention on chemical alterations of RNA nucleotides in the field of epitranscriptomics, this is about to change. In this review, we examine the chemistry of oxidatively-induced RNA damage, current analytical and mapping methods for analysis of oxidized nucleotides as well as their impact on biological processes.

The chemical similarities between DNA and RNA are reflected in many nucleobase oxidation products detected on both types of nucleic acids. This forms the basis for transposition of further results that have been elaborated with DNA and may reasonably be expected to apply to RNA as well. Consequently, this review focuses on studies performed on RNA, and will quote DNA-based results when transposition to RNA is warranted by the lack of concrete studies on the latter. Thus, while relatively similar behaviour can be expected at nucleobase level, the overall reactivity and oxidation biology of these two nucleic acids in the cell are substantially different. This is mostly owed to the classical differences in structural biology, but also relates to the much higher concentration of RNA in the cell. RNA's presence outside the nucleus also leads to generally higher exposure to various chemical reactions, undergone with endogenous reactive oxygen species or exogenous oxidants (Table 1).

**Table 1:** Structural and cellular features of DNA and RNA that modulate their susceptibility to oxidative damage

Feature	DNA	RNA
Structure	Double-stranded	Mostly single-stranded with double-stranded regions
Sugar	2′-Deoxyribose (no 2′-OH)	Ribose with 2′-OH
Protein protection/packaging	Compacted into nucleosomes by histones	Specific RNP complexes with RNA-binding proteins
Localization	Largely nuclear, but also present in mitochondria in eukaryotic cells (mtDNA)	Widely distributed: nucleus, cytoplasm, mitochondria, associated to ER membrane
Repair pathways	Multiple enzymatic repair systems (BER, NER, …)	Limited direct enzymatic repair
Turnover	Generally long-lived	Highly variable lifespan (from minutes to days)

Biological effects consequently relate to a variety of RNA species and locations. Impact on the abundant RNAs of the cellular machinery pertains to ongoing translation, and may lead to miscoding, defect translational machinery, and production of aberrant peptides. However, less abundant species involved in regulation, such as miRNA, are also known to affect signalling pathways upon oxidation. Overall, the diversity of biological effects of RNA oxidation is certainly still being underestimated considering the number of cellular processes involving RNA.

### Oxidative stress

**Fig. 1:**
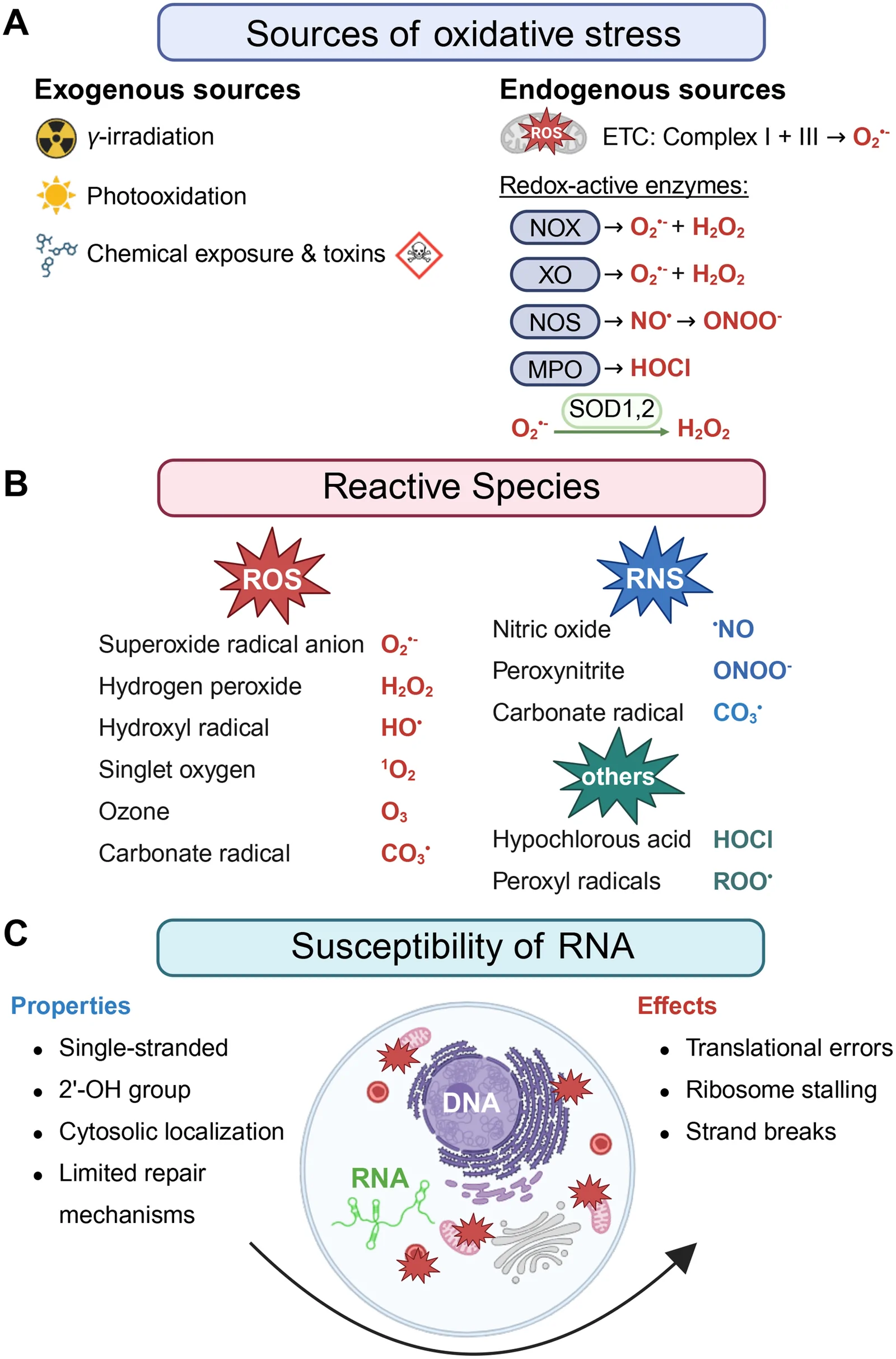
Sources of oxidative stress, major reactive species and RNA properties relevant to oxidation. (A) Major sources of oxidative and nitrative stress are grouped as exogenous (ionizing radiation, photooxidation and environmental chemicals) and endogenous (ETC and redox-active enzymes: NADPH oxidases NOX, xanthine oxidases XO, nitric oxide synthases NOS and myeloperoxidase MPO). These systems produce specific reactive oxygen (ROS) and nitrogen species (RNS) including superoxide radical anions O_2_˙^−^, H_2_O_2_, ˙OH, singlet oxygen ^1^O_2_, nitric oxide NO˙, peroxynitrite ONOO^−^ and hypochlorous acid. Superoxide dismutases (SOD1/2) convert O_2_˙^−^ into H_2_O_2_. (B) Classification of the major reactive species. (C) Properties of RNA and downstream effects of oxidative stress (red asterisks) acting on RNA in a cellular environment. Created in BioRender. Weber, Marlies. (2026) https://BioRender.com/d1qf8to.

From a chemical perspective, oxidative stress is defined as an imbalance between oxidizing and reducing agents present in the living cell (Fig. 1) and thus potentially leading to accumulation of various highly reactive entities. The maintenance of such redox equilibrium is pivotal for cellular homeostasis as both physiological oxidative signals (eustress) and excessive oxidative burden (distress) impact fundamental biological functions.^1–4^

Over the past decades, oxidative stress has attracted substantial attention due to its central role in numerous physiological and pathological processes. At physiological concentrations, reactive oxygen species (ROS) serve as essential signalling molecules in redox-regulated pathways.^1–3^ However, excessive ROS levels lead to untargeted oxidation of cellular components including lipids, proteins and nucleic acids.^2^ This transition from redox homeostasis to oxidative stress can be triggered by both exogenous and endogenous sources.^1,2,5–11^

Exogenous sources include ionizing radiation (*e.g.* γ-irradiation),^11^ photooxidation^12,13^ and exposure to environmental toxins or chemical agents.^14^ Endogenous ROS generation primarily occurs through physiological metabolic processes such as the mitochondrial electron transport chain (ETC)^3,4,7,15,16^ and through the activity of redox enzymes including NADPH oxidases (NOX).^1,9^ This oxidative load is further exacerbated in pathological conditions, namely chronic inflammation and degenerative diseases. Chronic inflammatory signaling can drive ROS accumulation by stimulating NOX enzyme activity and disrupting normal mitochondrial function. Meanwhile, the depletion of antioxidant defences limits ROS detoxification thus establishing a vicious cycle of oxidative stress and progressive cellular injury.^1,3,6,8,17^

The most physiologically relevant ROS include singlet oxygen (^1^O_2_), superoxide radical anion (O_2_˙^−^), hydrogen peroxide (H_2_O_2_), hydroxyl radicals (˙OH), carbonate radical (CO_3_˙^−^), and ozone (O_3_).^1,2,5,7–10,16,18,19^ Other contributors to oxidative stress include reactive nitrogen species (RNS), such as nitric oxide (NO˙) and peroxynitrite (ONOO^−^)^1,5,6,19^ hypohalous acids (HOX)^1,20–23^ and direct electron transfer in mitochondria.^1,4,15,16,24^

### Physiological sources of ROS and RNS

Cellular ROS and RNS originate from both enzymatic and non-enzymatic processes. A major endogenous source is the ETC, where complexes I and III leak electrons that reduce molecular oxygen to superoxide.^3,4,7,8,15,16^ Superoxide is rapidly converted by mitochondrial superoxide dismutase (SOD2) to H_2_O_2_, which although more stable, can generate different free radicals through Fenton chemistry in the presence of free Fe^2+^.^3,4,7,8,15,16^

For a long time ˙OH was considered as the most physiologically relevant free radical implicated in protein and nucleic acid damage. However, recent data indicate that it exists in the cells only under either X-ray exposure, or artificial non-physiological conditions (*e.g.* low pH). Under physiological pH (pH > 5), and the presence of Fe^2+^ ions, FeO_2_˙ (ferryl radical) is formed. In the presence of physiological concentrations of hydrocarbonate ions (HCO_3_^−^), this results in the formation of the carbonate radical (CO_3_˙^−^) as the major product.^25^ Taken together, under physiological conditions, and considering relative concentrations of Fe^2+^, CO_2_ and H_2_O_2_, the “Fenton” reaction in living cell produces CO_3_˙^−^ rather then ˙OH,^26^ and thus CO_3_˙^−^ is now emerging as a key mediator of oxidative RNA (and DNA) damage. Unlike the highly reactive and indiscriminate OH˙, CO_3_˙^−^ behaves as one-electron oxidant mostly in guanine oxidation in DNA and RNA, due to its redox potential (see Fig. 3).^26^ Although generation of CO_3_˙^−^ contributes to lesion formation, bicarbonate/CO_2_ also exerts a protective effect^27^ by diverting the cellular outcome of Fenton reaction from highly reactive ˙OH to CO_3_˙^−^, thereby attenuating iron-catalyzed nucleic acid oxidation in both bacterial and human cells.^28^ Consequently, the balance between CO_3_˙^−^-driven damage and CO_2_-mediated antioxidant capacity shapes the oxidative fate of RNA under physiological and stress conditions. Recognition of this dual role will be crucial for interpreting RNA-damage assays, understanding the contribution of radical chemistry to disease-related chemical derivatives of canonical RNA, and potentially designing interventions that exploit the carbonate system to mitigate oxidative injury.

Furthermore, H_2_O_2_ also serves as a substrate for myeloperoxidase (MPO) forming hypohalous acids such as hypochlorous acid (HOCl) in neutrophils.^1,3,19,29^ Hypochlorous acid is highly reactive and damages a broad spectrum of biomolecules. Oxidation of lipids and proteins further amplifies ROS generation by producing peroxyl (ROO˙) and alkoxyl (RO˙) radicals that initiate radical chain reactions resulting in the formation of non-functional molecules and damaged membranes.^1–3,10,20,21^

Moreover, several redox-active enzymes significantly contribute to intracellular and extracellular ROS and RNS production, especially under inflammatory and metabolic stress conditions. NADPH oxidases (NOX) are membrane-bound enzymes that generate superoxides as part of the immune response. While NOX-derived ROS defend against pathogens, they also contribute to oxidative damage in inflamed or metabolically stressed tissues.^1,6,9^ Xanthine oxidases (XO) convert hypoxanthine to xanthine and uric acid, thereby constantly producing superoxide and H_2_O_2_ as by-products. XO activity increases under ischemia or inflammation leading to ROS accumulation.^1,6,8,30^ Nitric oxide synthases (NOS) produce nitric oxide (NO˙) as a signalling molecule, but it readily reacts with superoxide to form peroxynitrite, known to be a powerful oxidant of nucleic acids.^1,5,6,8,19,31^

Myeloperoxidase (MPO), highly expressed in neutrophils and monocytes, utilizes H_2_O_2_ and halide ions (mainly Cl^−^) to generate HOCl. Though essential in antimicrobial defense, MPO activity contributes to host RNA and protein oxidation during chronic inflammation.^2,6,20–22,32,33^

In addition, there are several biologically relevant systems that facilitate the transfer of energy to oxygen, thereby generating singlet oxygen. Physiological examples include peroxidases (*e.g.* myeloperoxidase) and oxygenases (*e.g.* lipoxygenases) and light-dependent mechanisms involving photosensitizers which are excited upon UVA radiation.^34^

Collectively, these enzymes constitute an essential system for the regulated generation of ROS/RNS in immune defense and redox signalling. However, under imbalance conditions, these ROS/RNS contribute to oxidative damage of biomolecules such as RNA.

To counteract oxidative stress, cells employ sophisticated antioxidant defense systems consisting of both enzymatic and non-enzymatic components. These include small molecule antioxidants such as glutathione (GSH), NADPH and vitamin C and redox-active enzymes including superoxide dismutases (SOD1 and SOD2), catalase (CAT) and glutathione peroxidases.^2,6,10^ The molecular mechanisms of oxidative stress defence are extensively reviewed elsewhere^8,35–42^ and will not be considered in detail here.

## Susceptibility of RNA to oxidative damage

While all biomolecules are potential targets of oxidative stress, RNA is particularly vulnerable to oxidative damage due to its structure and subcellular location. Although RNA and DNA share the same canonical nucleobases (A, C and G), a sugar-phosphate backbone and base pairing properties, RNA is mostly single-stranded, lacks protective histone complexation and is primarily localized in the cytosol near mitochondria and the endoplasmic reticulum, which are both major sites of ROS generation.^43,44^ RNA damage is further amplified by its continuous exposure to oxidative environments during transcription, processing and translation. Unlike DNA, sophisticated and complex repair mechanisms for RNA have not been described (yet), even if some oxidized RNA bases can be removed.^45^ Instead, cells rely mostly on surveillance pathways, such as no-go decay or nonsense-mediated decay, to identify and degrade oxidized RNA transcripts.^43,46^ MTH1 and NUDT5 are two important players involved in recognition and degradation of oxidized purine nucleoside triphosphates (NTPs), such as 8-oxo-GTP/GDP. These enzymes facilitate the hydrolysis into monophosphates, thereby preventing their accidental incorporation into RNA.^43,44,47–51^ Oxidative lesions, especially 8-oxoguanosine (oxo^8^G) have been observed to accumulate even under basal conditions and interfere with translation fidelity, potentially causing ribosome stalling or synthesis of misfolded proteins.^43,44,51^

The following chapters of this review will discuss the broader biological significance of RNA oxidation as well as the chemical features of oxidized RNA and current detection methods.

## Chemistry of oxidative RNA damage

**Fig. 2:**
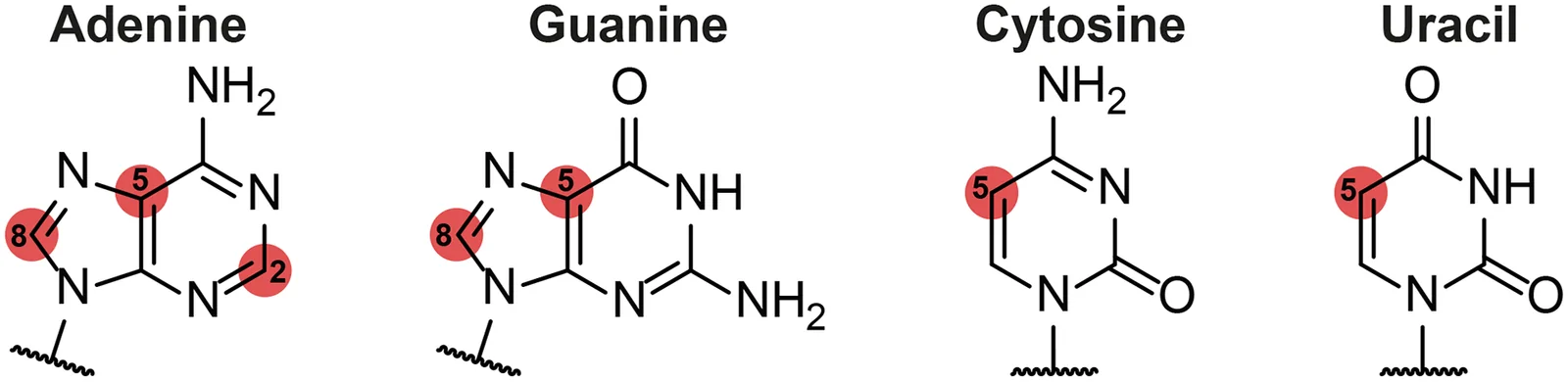
Reactive sites for nucleobase oxidation in RNA. Red circles mark the atom most commonly targeted by oxidants. Purines (adenine and guanine) are primarily oxidized at C8 and C5 carbon atoms. Adenine additionally undergoes oxidation at C2. Pyrimidines (cytosine and uracil) are predominantly oxidized at the C5 position. The figure shows base atoms only, ribose oxidation sites are not depicted.

In RNA molecules, oxidation primarily affects the nucleobase (Fig. 2)^52–55^ with guanosine base being the most susceptible, based on its redox potential *E*^0^(*E*^0^(G) = 1.29 V), followed by adenosine (*E*^0^(A) = 1.42 V), cytidine (*E*^0^(C) = 1.6 V) and uridine (*E*^0^(U) = 1.72 V).^56^ This order represents the relative ability of electron abstraction under oxidative conditions, particularly in the presence of ROS like cabronate radicals. The most important oxidation products in RNA include 8-oxoguanosine (oxo^8^G),^51,57^ 8-oxoadenosine (oxo^8^A),^57,58^ 5-hydroxycytidine (ho^5^C) and 5-hydroxyuridine (ho^5^U),^53–55^ with oxo^8^G being by far the most abundant and thus the focus of numerous studies.^51^ Oxidized purines in DNA, but also in RNA, may undergo to spontaneous depurination yielding abasic (apurinic/apyrimidinic) sites (rAP) that critically disturb the integrity and structure of RNA. In addition to the nucleobase, oxidation can also occur on the ribose-phosphate backbone, potentially forming strand breaks. Furthermore, most of these oxidized nucleosides have altered distribution of H-bonds donors and acceptors, thus compromising base-pairing properties that are essential for RNA–RNA and RNA–protein interactions.^52,59,60^

The findings reported in this chapter are mainly based on studies performed on free deoxyribonucleosides or DNA. But as these investigations deal with the molecular level of oxidation reactions, we have included them to cite our statements.^61–64^

### Oxidation of guanosine: mechanisms, products and biological implications

**Fig. 3:**
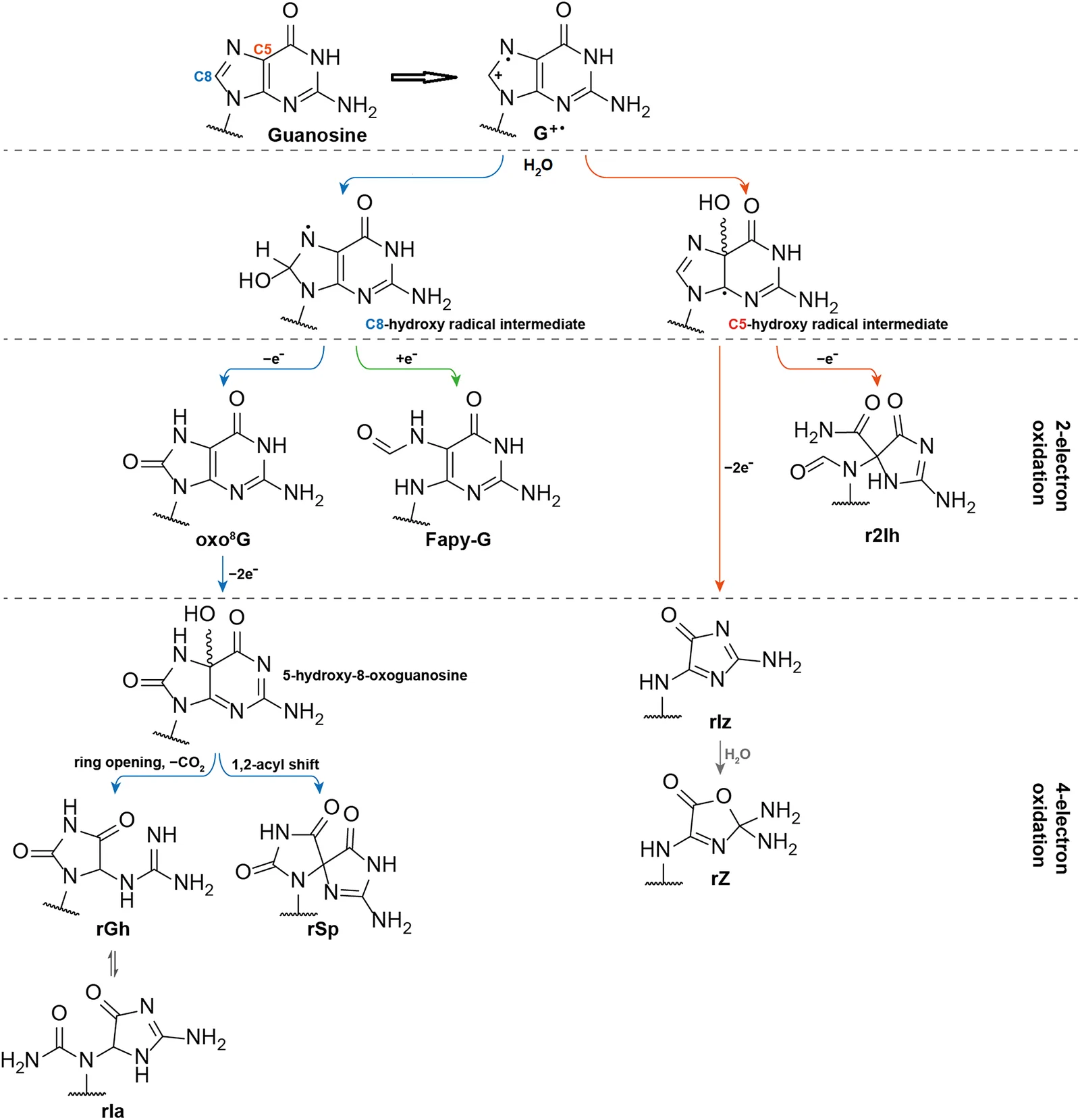
Oxidation pathways and principal products of guanosine. C8-directed oxidation (blue arrows) leads to 8-oxoguanosine (oxo^8^G), which is further oxidized to the hydantoins guanidinohydantoin (rGh) and spiroiminodihydantoin (rSp). rGh can isomerize into ribo-iminoallantoin (rla). Under reducing conditions, the same C8 radical intermediate can be also reduced and ring-opened to yield formamidopyrimidine (Fapy-G, green arrow). C5-directed oxidation (orange arrows) leads to the two-electron product 5-carboxamido-5-formamido-2-iminohydantoin (r2Ih) and to the four-electron oxidation products imidazolone (rIz) and its hydrolyzed counterpart oxazolone (rZ). Reaction outcomes strongly depend on oxidant identity, pH, redox environment and local nucleic acid structure.

Among the four canonical ribonucleosides, guanosine (G) is the most susceptible to oxidative modifications due to its low redox potential.^56^ Oxidation mainly targets the C8 and C5 positions of the purine ring, producing a diverse set of chemical lesions. These include 8-oxoguanosine (oxo^8^G), spiroiminodihydantoin (rSp), guanidinohydantoin (rGh), iminoallantoin (rIa), 5-carboxamido-5-formamido-2-iminohydantoin (r2Ih), imidazolone (rIz), oxazolone (rZ) and formamidoguanosine (Fapy-G) (see Fig. 3).^65,66^

### C8-directed oxidation pathways

8-Oxoguanosine, the most widespread guanosine oxidation product, arises from a two-electron oxidation at the C8 position. It is a well-known marker of oxidative RNA damage and is also found as an abundant lesion in DNA.^51,67^ With a redox potential of ∼0.6 V (compared to 1.29 V of G), oxo^8^G is more susceptible to oxidation than any of the canonical bases, leading to a variety of secondary oxidation reactions, the outcome of which depends on the reaction conditions.^56,57,62,68^

Formation of oxo^8^G in RNA can be triggered by a wide range of ROS, including H_2_O_2_, ˙OH, CO_3_˙^−^ and UV-induced photooxidation.^57,64,69–73^ At the molecular level, G oxidation to oxo^8^G may proceed *via* direct electron abstraction at C8, followed by H_2_O addition (Fig. 3). Oxo^8^G exhibits particular chemical and structural properties that have important biological implications. It is prone to further oxidation producing downstream lesions such as rGh and rSp. Its generation occurs at the nucleotide level and in nucleic acid. The lesion induces a conformational change from *anti*- to *syn*-enabling Hoogsteen base-pairing with adenosine or cytidine.^51,52,57,74–77^ Compared to many other oxidative guanosine lesions, oxo^8^G accumulates *in vivo* to detectable levels in all reported investigations.^45,46,78,79^

A comprehensive review on oxo^8^G in DNA and RNA including its *in vivo* occurrence, biological effects and mechanisms for elimination of oxo^8^G-containing mRNA has been recently published.^51^ Sustained oxidative stress drives further oxidation of oxo^8^G producing rGh and rSp through a shared 5-hydroxy-8-oxoguanosine intermediate (see Fig. 3).^64,68,80^ The relative abundance of rGh and rSp depends on multiple factors including pH, oxidant type and the structural context of the guanosine residue.^61,63,64,66,68,70,80–84^ rSp predominates under neutral to alkaline conditions, while rGh formation is favored in acidic environments and duplex structures following ring opening and decarboxylation.^63,64,68,80^ Furthermore, rGh can reversibly tautomerize into iminoallantoin (rIa) under mildly alkaline conditions (pH ∼ 8.2), though its biological relevance remains under discussion.^61,63,80,82,83^

### C8 oxidation in the presence of reductants: Fapy-G formation

The formamidopyrimidine derivative of guanosine (Fapy-G) forms under oxidative stress through ˙OH or CO_3_˙^−^ radicals^57,69,85–89^ or ionizing radiation^64,88,90^ in the presence of reductants.^64,85,90–92^ Its formation begins with a one-electron oxidation at the C8 of guanosine generating the same C8-OH radical intermediate that precedes 8-oxoguanosine (oxo^8^G) formation as depicted in Fig. 3. In the oxo^8^G pathway, this intermediate undergoes a second one-electron oxidation, whereas in the Fapy-G pathway it is reduced, triggering imidazole ring opening followed by hydrolysis, to yield the formamidopyrimidine structure. Both lesions share the same radical intermediate making their formation competitive. Reductive conditions favor Fapy-G, whereas more oxidizing environments shift the product distribution toward oxo^8^G. Fapy-G is chemically labile, particularly to acid hydrolysis and elevated temperatures, making its detection challenging.^64^ This instability may contribute to variable reports on its occurrence under physiological conditions, although it remains sufficiently stable at physiological pH and temperature and when embedded within nucleic acids.^64,69,85–88,90–92^ As stated previously, the literature cited in this paragraph refers to DNA. However, it is important to note that Fapy-G has been directly detected in tRNA following UV irradiation^93^ and in rRNA exposed to ˙OH generated by Fenton chemistry^69^ confirming that this lesion is also relevant for cellular RNA under defined oxidative conditions.

### C5-directed oxidation pathways

C5-directed oxidation of guanosine gives rise to r2Ih (Fig. 3), a hydantoin lesion formed under conditions such as ozone exposure^94,95^ or ˙OH stress.^64,91,96,97^ The formation of r2Ih was first described as a direct guanine oxidation product in DNA.^98^ r2Ih results from epoxidising oxidative pathways, which can occur *in vivo* under conditions of elevated lipid peroxidation or during inflammatory responses that produce organic peroxides and related reactive species.^98^ Several studies have proposed that the r2Ih is as prevalent as oxo^8^G under conditions similar to cellular systems. Nevertheless, due to its instability in the form of the isolated nucleoside, data concerning its real abundance in RNA remain limited. However, r2Ih generated in the RNA chain by oxidation, may be more stable, implying correspondingly more potential relevance in certain cellular settings.^64,95^

An alternative oxidation pathway targeting the C5 position of guanosine leads to the formation of imidazolone (rIz) and its hydrolysis product oxazolone (rZ) (Fig. 3). These lesions are considered as minor products of nucleoside oxidation and are typically formed under conditions that lack reductants favoring a perhydroxylation mechanism rather than the hydroxylation route that yields r2Ih.^64,91,96^ Their formation has been well-documented in DNA and ribonucleoside model systems, but evidence for their presence in structured RNA remains limited. The oxidation pathway of rIz and rZ is highly sensitive to oxidant type, reaction conditions (*e.g.* pH, buffer composition) and the presence or absence of reductants. These factors collectively determine whether r2Ih or rIz/rZ predominates.^96^ The perhydroxylation step is a distinguishing chemical feature of rIz formation and suggests that oxidant specificity plays a pivotal role in the reaction outcome. For example, ˙OH radicals and epoxidizing agents such as peracids can drive rIz and rZ formation in DNA, and analogous mechanisms are chemically plausible for RNA.^91,96^ Although direct confirmation of rIz and rZ presence in RNA is lacking, the fundamental chemical feasibility of their formation is supported by oxidation studies on ribonucleosides and clear identification of such lesions in DNA.^82,91,96,97,99–108^ Despite their plausible formation, rIz and rZ are generally minor products compared to more abundant lesions like rGh, rSp and r2Ih, particularly under physiologically relevant redox conditions. In addition, their stability, mutagenicity and their downstream biological effects in RNA remain underexplored. Therefore, further studies are necessary to determine their real occurrence in cellular RNA and to elucidate any functional consequences that may arise from their presence.

### Nitrative RNA lesions

Reactive nitrogen species (RNS) are produced in inflamed tissues particularly *via* inducible nitric oxide synthase (iNOS). One major RNS is peroxynitrite (ONOO^−^), which, can generate various secondary radicals and reactive species, depending on the pH and the presence of CO_2_. Under physiological pH, peroxynitrite reacts with CO_2_ and decomposes to yield the carbonate radical CO_3_˙^−^ and nitrogen dioxide radical (NO_2_˙). Upon protonation to ONOOH, decomposition results in HO˙ and NO_2_˙.^6,31^ These secondary radicals drive both oxidative and nitrative damage at guanosines. CO_3_˙^−^ is a potent one-electron oxidant that promotes formation of oxo^8^G and downstream hydantoins, whereas NO_2_˙ gives mainly rise to 8-nitroguanosine (8-NO_2_-G).^62,109–116^ Moreover, secondary nitrosative agents generated upon ONOO^−^ decomposition^117^ promote A, C and G deamination, resulting in A to I and C to U tansitions.^118^ The relative abundance of RNS-related oxidation products is highly sensitive to local reaction conditions (CO_2_/bicarbonate, pH and the presence of reductants or metal catalysts).^62,115^ Importantly, 8-NO_2_-G is readily generated *in vitro* and in cells and appears comparatively stable in RNA (unlike 8-NO_2_-dG in DNA) making it a useful marker for nitrosative stress in RNA studies.^62,109,111,112^

### Photooxidation of guanosine

Photooxidation proceeds through two principal mechanisms: Type I, involving direct electron transfer and hydrogen/proton abstraction from an excited photosensitizer and Type II, in which the photosensitizer transfers energy to molecular oxygen generating singlet oxygen (^1^O_2_).^34,119^ In the Type II pathway, ^1^O_2_ reacts directly with guanosine to form a transient 4,8-endoperoxide, which undergoes rearrangement and reduction to yield oxo^8^G, the major oxidation product identified in DNA.^34,120^ oxo^8^G can be further oxidized into secondary lesions such as spiroiminodihydantoin (rSp), imidazolone (rIz) and oxazolone (rZ) under continued oxidative stress.^34,62,83,103,121–125^ The balance between Type I and Type II mechanisms strongly depends on reaction conditions such as pH, local environment, the nature of the photosensitizer and nucleic acid structure, which explains the variability in reported product distributions and the ongoing debate regarding their relative abundance. While most mechanistic data come from DNA and model nucleosides, the same chemical principles apply to RNA as well.^34,62,83,122^

### Occurrence of RNA guanosine lesions in biological systems

Biological studies confirm that multiple guanosine lesions arise in cellular RNA under oxidative and nitrative stress.^69,70,93^ For example, oxo^8^G, rGh and Fapy-G were detected in *E. coli* ribosomal RNA following UVA exposure and Fenton-induced oxidative stress in the absence of bicarbonate and very high H_2_O_2_ concentration.^69^ Notable differences were observed between isolated rRNA and ribosome-associated rRNA, with rGh predominating after UVA and Fapy-G after Fenton treatment. Similarly, rGh was reported as the principal lesion of UV-damaged tRNAs, particularly at solvent-exposed guanosines, indicating that RNA structure modulates susceptibility to oxidation.^70,93^ In parallel, 8-NO_2_-G has been detected *in vivo* and is widely used as a marker of nitrative stress associated with inflammation and cancer.^109,111–113^

Many of these lesions alter native guanosine's base-pairing properties,^63,75,76,103,126–128^ potentially compromising RNA structure, translation and function. Despite the great relevance of guanosine oxidation products in the disease context (see below in the Chapter “Biological consequences of RNA oxidation”), the identification and quantification of these molecules remain technically challenging due to their chemical instability, limited UV absorbance, propensity for hydrolysis and spontaneous isomerization.^64^

### Adenosine oxidation reactions

**Fig. 4:**
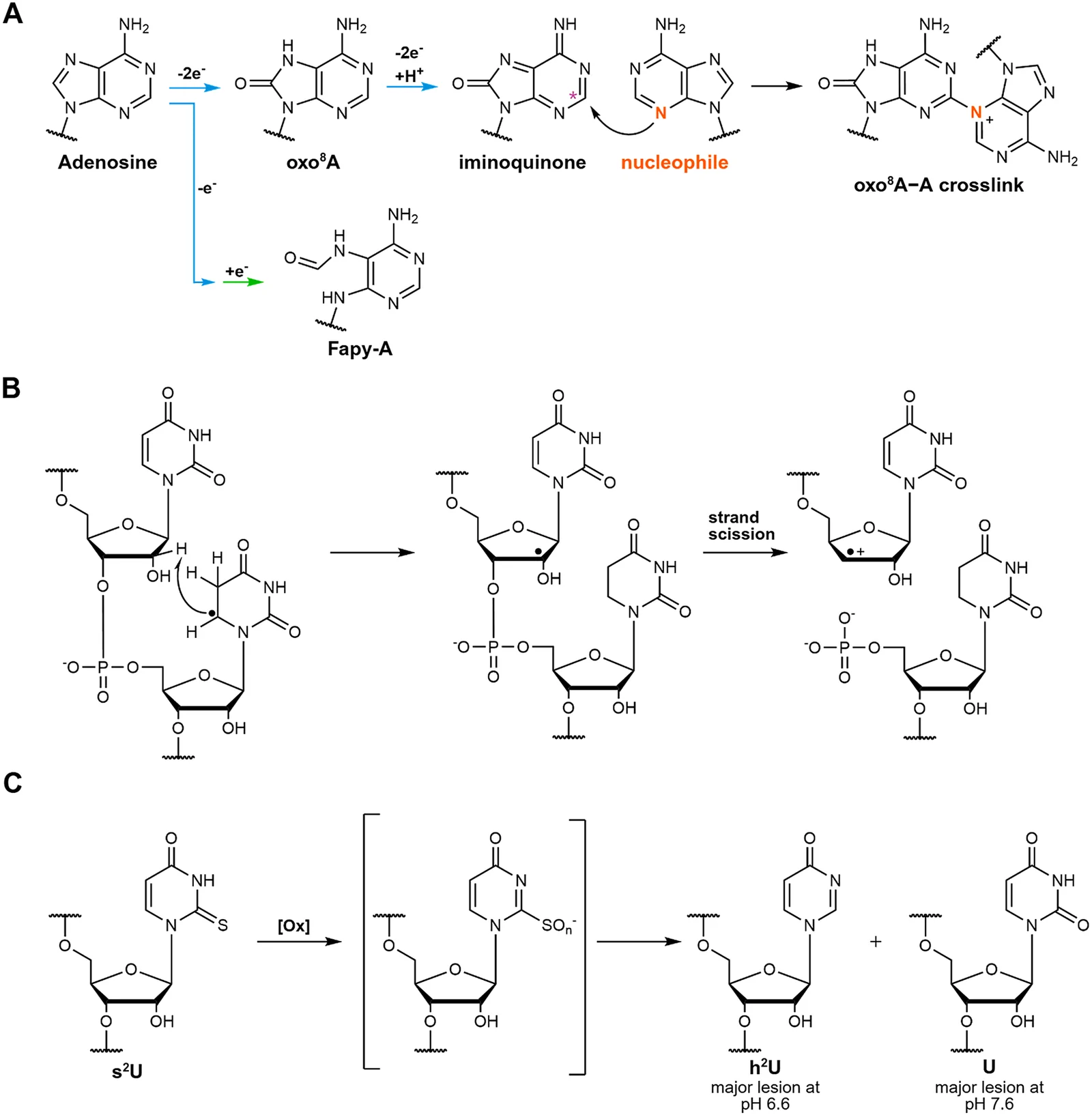
(A) Cross-link formation between 8-oxoadenosine (oxo^8^A) and adenosine following iminoquinone formation. Primary oxidation of adenosine generates oxo^8^A, which can undergo a subsequent oxidation step to form the short-lived iminoquinone intermediate. The C2 position of this electrophilic species (purple asterisks) is highly reactive toward nucleophiles including ring nitrogens of nearby purines (orange), thereby promoting intermolecular cross-link formation. Fapy-A is an alternative oxidation product formed by a consecutive reduction of the radical intermediate following initial oxidation. Blue arrows indicate oxidation, green arrows indicate reduction and black arrows indicate cross-link formation. (B) Proposed mechanism of radical uridine oxidation-mediated strand scission in RNA. A radical at the C6 atom in U residue attacks C2′ atom in the upstream (5′) RNA residue, leading to strand break. (C) Oxidation of naturally occurring 2-thiouridine (s^2^U) residues in tRNAs. Oxidation of the sulfur can lead to h^2^U or canonical U residues, depending on the pH of the medium. A carbene-intermediate was proposed for the de-modification (in fact a reductive removal of the s^2^-group), which preferentially occurs at physiological pH in tRNA.^132^ Created in BioRender. Weber, M. (2026) https://BioRender.com/7e7ek3y.

Compared to guanosine, adenosine is less susceptible to oxidative damage due to its higher redox potential (*E*^0^_A_ = 1.42 V). One-electron oxidation of A residue in DNA^129^ was shown to result in charge transport to nearby G, similar reactions take place also in RNA. Direct oxidation at the C8-position yields 8-oxoadenosine (oxo^8^A), which displays a significantly lower redox potential 
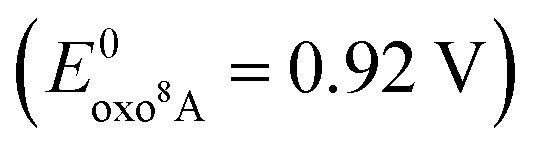
 and therefore becomes highly prone to further oxidation.^58,59,69,130^ Unlike oxo^8^G, secondary oxidation reactions at oxo^8^A result in a quinoidal iminoquinone intermediate that is uniquely characteristic of adenosine chemistry (Fig. 4A). This highly electrophilic species reacts at the C2 position with nucleophiles including water, exocyclic amines, lysine, histidine and cysteine residues leading to the formation of covalent adducts as RNA-protein crosslinks.^57,58,130,131^ In addition, formamidopyrimidine derivatives of adenosine (Fapy-A) can form when the C8-OH radical intermediate is reduced instead of further oxidized. Thus, as for guanosine, the balance between oxo^8^A and Fapy-A strongly depends on the local redox environment, with reductive conditions favoring Fapy-A. While adenosine oxidation has been studied less extensively than for guanosine, this unique quinoidal pathway underscores potentially distinct biological consequences of oxidative stress on RNA.^24,57,69,70^

### Oxidative damage to pyrimidines

In contrast to extensive data available on purine oxidation, research on pyrimidine oxidation in RNA is relatively sparse. In addition to oxo^8^A and oxo^8^G, 5-hydroxycytidine (ho^5^C) and 5-hydroxyuridine (ho^5^U) were identified in *in vitro* oxidoreduction experiments conducted in the presence of NADH and K_3_Fe(CN)_6_ in *Torula* yeast RNA.^53,54^ Additionally, numerous other oxidation products originating from ROS have been identified, particularly in the case of cytosine in DNA. Furthermore, cytidine forms a stable chlorination product upon oxidation with HOCl. 5-Chlorocytidine (Cl^5^C) has been detected in concentration-dependent HOCl oxidations, side-by-side with 5-chlorouridine (Cl^5^U) and the corresponding hydroxy-modified pyrimidines, ho^5^C and ho^5^U.^133^ Moreover, Cl^5^C was formed from HOCl that was enzymatically generated of H_2_O_2_ and Cl^−^ by myeloperoxidase (MPO).^134,135^

### Oxidation of the ribose sugar and strand breaks

In addition to base oxidative damage, the ribose in the RNA chain is also a target for oxidation and seems to be even more sensitive, compared to the respective DNA sugar.^136^ Radical oxidation of the sugars in DNA and RNA was shown to be mediated by nearby pyrimidine residues. Exposure of DNA or RNA to ionizing radiation leads to formation of cation radicals, followed by their hydration and secondary reaction with C2′ atom in the ribose (desoxyribose) leading to direct strand breaks at the 5′-adjacent nucleotide. Such strand scission occurs preferentially in double stranded RNA (Fig. 4B) and in the absence of molecular oxygen (O_2_).^137,138^ Interestingly, such oxidative breaks in RNA generate 5′-P residues, unlike commonly observed nuclease-mediated RNA degradation.

### Interplay of chemical oxidation with natural RNA modifications

Some enzymatic RNA modifications, particularly sulfur-containing derivatives, exhibit increased susceptibility to form secondary modification products when exposed to chemical oxidation. Thiouridine derivatives (s^2^U, s^4^U and mnm^5^s^2^U) are well-documented to undergo oxidative desulfurization (Fig. 4C). For instance, oxidation of s^2^U with exogenous or endogenous H_2_O­_2_ generates 4-pyrimidinone ribofuranoside (h^2^U) *in vivo* and *in vitro*.^59,139–141^ This reaction is pH-dependent: at acidic pH (6.6) H^2^U is predominantly formed, while at higher pH (7.6) the main product is uracil.^139,141^ Similarly, mild oxidation of s^4^U with permanganate leads to the formation of uridine 4-sulfonate as an intermediate and subsequently reversion back to uridine.^142^ Another 2-substituted derivative of uridine, 2-selenouridine (Se^2^U) is even more susceptible to oxidation. In the first step, Se^2^U is oxidized to the corresponding diselenide (Se^2^U)_2_, an unstable intermediate that subsequently decomposes to uridine and selenium.^59,143^ Further oxidation products have been described for tRNA modifications,^70,144,145^ but despite these scattered findings, the behavior of natural RNA modifications in RNA damaging conditions and the associated biology remain largely unexplored.

### Abasic sites in RNA – a chemical perspective

**Fig. 5:**
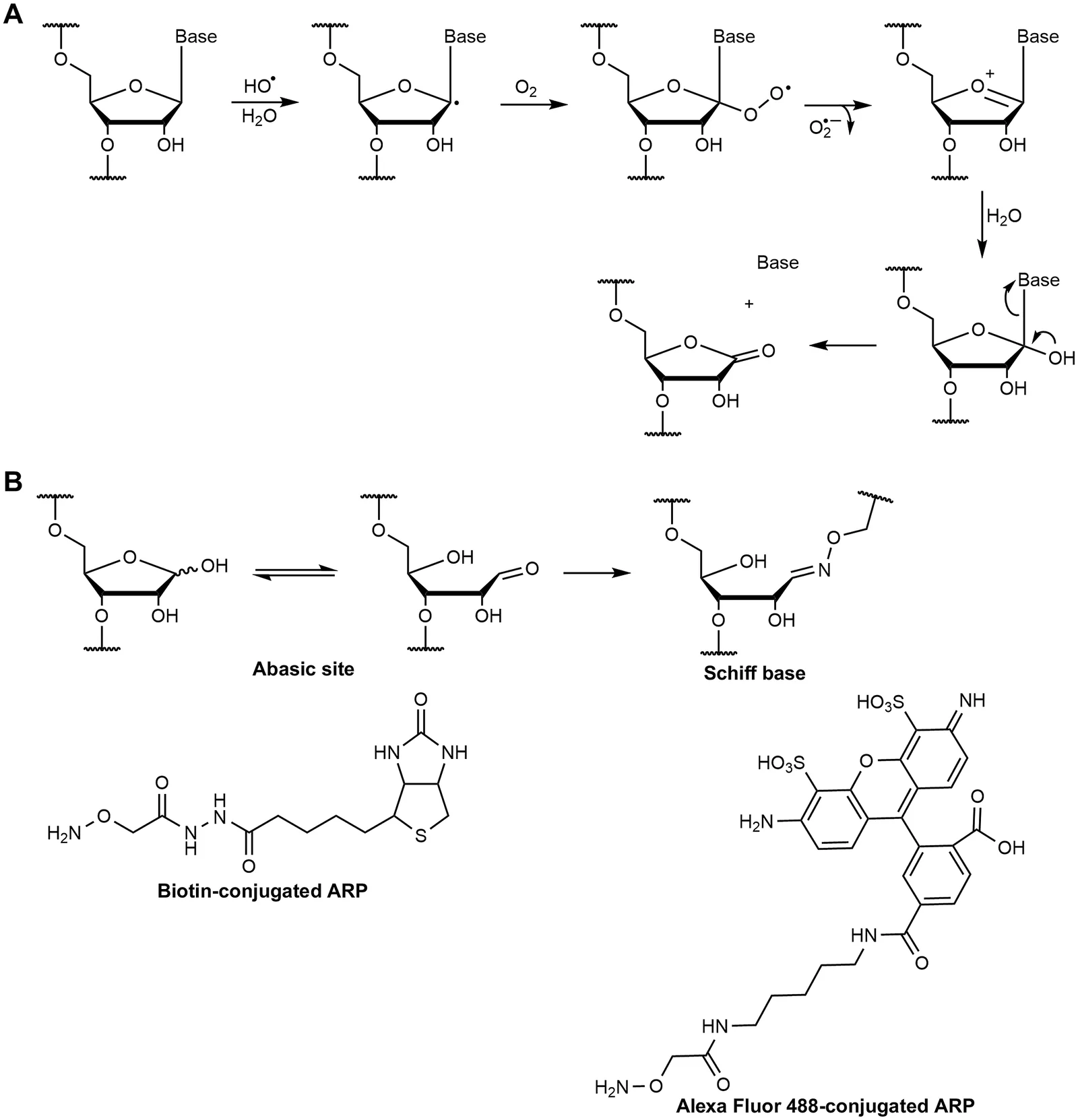
Abasic sites formation under oxidative stress and their susceptibility to nucleophilic attack. (A) Oxidative stress promotes depurination *via* C1′ oxidation generating lactone abasic sites with a reactive aldehyde at the ribose C1′ position. (B) Hemi-aminals abasic sites exist in equilibrium between an open-ring aldehyde and a closed semiacetal form. The aldehyde is highly prone to nucleophilic attack by primary amines resulting in imine (Schiff base) formation. Potential amines include free amino acids, proteins, or hydroxylamine moiety, commonly used in tagging reagents containing biotin or fluorescent dyes. Figure adapted from Prashar *et al.* 2023.^147^ Created in BioRender. Weber, M. (2026) https://BioRender.com/6mn3b21.

rAP sites in RNA are formed when the *N*-glycosidic bond at the ribose anomeric carbon is cleaved, releasing either an intact or an oxidatively-modified base and leaving a ribose only moiety (AP ribose). A ribose radical intermediate at C1′ promotes base elimination *via* an oxocarbenium-type cation (Fig. 5A), after hydration leading to the final formation of oxidized rAP site (lactone structure).^78,146,147^ Other oxidative routes to hemi-aminals rAP sites (Fig. 5B) typically follow base oxidation: radical attack (electron abstraction, ˙OH, lipid peroxidation) or photooxidation producing destabilized, oxidized nucleosides such as oxo^8^G. Purine residues, in particular guanosines, are more prone to yield rAP sites because their *N*-glycosidic bonds are naturally weaker and become more labile after electron withdrawal by prior base oxidation.^146–148^ Chemically, the hemi-aminals rAP ribose exists in a dynamic equilibrium between the closed furanose hemiacetal form and the open-ring aldehyde form Fig. 5B, which is susceptible towards nucleophilic attacks, resulting in the formation of a Schiff base. This oxime adduct facilitates intramolecular β-elimination ultimately leading to strand scission, but also crosslinking to nucleophilic side chains in proteins. Cytochrome *c*-catalysed oxidation within the mitochondrial respiratory chain can induce rAP site formation and promote RNA–protein crosslinks linking the chemical lesion to biological stress signalling in mitochondria.^146^ Detection studies using aldehyde reactive probes (ARP) further confirmed that oxidatively damaged RNA accumulates abasic sites, consistent with ribose oxidation and base detachment under radical attack.^72,149^ The distinct reactivity of RNA abasic sites can be utilised in NGS-based methods, such as OxiAbSeq.^133^ This method enables transcriptome wide detection of abasic sites through aldehyde-dependent strand scissions. The intrinsic instability of rAP sites complicates detection, contributes to variability among laboratories, and is likely the reason why reported abasic site levels vary widely depending on oxidant type, pH, and sample preparation.^147^ Nevertheless, compared to DNA, abasic sites in RNA are sufficiently stable under physiological pH and temperature to persist in nucleic acid polymers, making them chemically plausible lesions *in vivo*.^150^

### Particular chemistry of HOCl-induced oxidation of RNA

Hypochlorous acid (HOCl) is naturally produced by the enzyme myeloperoxidase (MPO) present in neutrophils and macrophages through oxidation of chloride ions by H_2_O_2_ during immune response. At physiological pH, HOCl (p*K*_a_ = 7.46) coexists with its conjugate base OCl^−^, but HOCl is the more potent oxidant (*E*^0^ = 1.48 V *vs.* 0.9 V) and freely diffuses across membranes. In the cellular environment, HOCl generates secondary oxidants such as ˙OH, superoxide radical anions and chlorine radicals, depending on pH, chloride concentrations and UV exposure.^20,21^ HOCl reacts with nucleic acids *via* chlorination and oxygenation of nucleobases at the C8 position of purines and the C5 position of pyrimidines resulting in the chlorinated nucleosides: 8-chloroguanosine (Cl^8^G), 8-chloroadenosine (Cl^8^A), 5-chlorocytidine (Cl^5^C) and 5-chlorouridine (Cl^5^U) as well as the corresponding oxygenated nucleosides. Furthermore, chlorination can also occur on heterocyclic and exocyclic nitrogens, but these chloramines showed only limited stability.^21,116^ The set of formed products strongly depends on reaction conditions and can impair transcription and translation. 5-Chlorocytidine (Cl^5^C), 8-oxoadenosine (oxo^8^A) and 8-oxoguanosine (oxo^8^G) were detected in RNA after oxidation using HOCl or *in vitro* myeloperoxidase systems.^32,109,116,130,151–153^ Many chlorinated RNA products were also linked to inflammatory diseases.^32,116,151–154^

## Analytical methods for detection, quantification and mapping of RNA oxidated/damaged nucleotides

The primary methods for direct detection of RNA oxidation in general as well as particular oxidized or damaged RNA residues are based on a handful of techniques from instrumental analysis. These include protocols of UV/Vis spectroscopy, chromatographic separation (TLC and column chromatography) and mass-spectrometry. More specific approaches exploit particular reactivities and biochemical properties of oxidized bases, nucleosides and nucleotides. Those include antibody-based enrichment and detection, aldehyde reactive probes (ARP), specific intercalating probes, and analysis of reverse-transcription signatures induced by damaged nucleotides. More recently, methods based on next generation sequencing of nucleic acids (both second (NGS) and third generation (NNGS, next–next generation sequencing) protocols) have also been adapted for sensitive and nucleotide-resolution detection.^51,69,72,116,155^ These techniques are often used in different combinations to enhance detection specificity and precision.

### Thin-layer chromatography (TLC)

Non-canonical nucleotides in RNA (as well as DNA) are amenable to analysis by TLC, subsequent to complete digestion of the RNA chain to the respective mononucleotides (NMPs or dNMPs). After digestion, the 5′- or 3′-phosphate of RNA may provide sensitivity, when labelled by radioactive ^32^P, either before (pre-labeling) or after (post-labeling) digestion to NMPs/dNMPs.^156,157^ For analysis of NMP composition, classical and general methods for the detection of RNA modifications can be employed, for instance thin-layer chromatography (TLC)^158^ in mono- (1D) or two-dimensional (2D) setup, using PEI^156^ or cellulose TLC plates.^159^ The optimization of stationary and mobile phases for the separation of mononucleotides has been extensively studied in the past to achieve the best resolution and separation of various RNA modifications. Now, established reference maps are available for commonly found naturally modified residues.^160^ These methods were initially used to detect enzymatically modified RNA nucleotides,^161,162^ but can also be extended to study RNA damage. TLC-based methods are simple to use, provide very high sensitivity, especially when coupled to ^32^P labeling and are relatively affordable. However, complete digestion to NMPs leads to an inherent loss of position-specific information. Without ^32^P labeling, sensitivity is modest or low. Additionally, the exact nature of spots appearing in 1D or 2D TLC profiles should be confirmed by independent, orthogonal approaches.

### Classical column chromatography and high-performance liquid chromatography (HPLC)

Analysis and preparative isolation of modified nucleosides and nucleotides by column chromatography was first described in the early 1970s^163^ and the analysis of oxidized G derivatives in DNA and RNA was also reported.^164,165^ Modified RNA (or DNA) nucleosides are separated upon complete hydrolysis of RNA (DNA) to NMPs (dNMPs). For RNA analysis, this approach involves enzymatic cleavage of the RNA chain into a mixture of nucleosides. Endonucleases (typically nuclease P1 and phosphodiesterase) and phosphatases are used in a combined treatment to cleave the RNA chain and dephosphorylate the 5′-end at the resulting NMPs to nucleosides.^166,167^ Subsequently, the resulting nucleosides are subjected to HPLC separation on a reversed-phase column (generally RP-18) using a binary mobile phase consisting of aqueous ammonium acetate and pure acetonitrile (or methanol). Elution is based on the hydrophobicity of the nucleosides, leading to characteristic retention times detected by UV-absorption or electrochemistry. Variations of this method have been extensively used to characterize various DNA and RNA photoproducts, including oxo^8^G.^168,169^ Recently, these protocols have also improved in terms of speed and sensitivity of detection.^71,170^ Liquid chromatography can be replaced by capillary electrophoresis coupled to electrochemical detection of modified nucleosides for separation.^171^ Commercial synthetic nucleosides are available as standards for external calibration. Alternatively, internal standards can be introduced *via* isotope labeling using ^13^C or ^15^N. Sensitivity and reproducibility of HPLC methods are generally high and coupling with MS (or MS/MS) analysis is straightforward and provides additional advantages (see below). However, these chromatographic methods are not high-throughput, and require complex data treatment and calibration for quantitative results. As with TLC, any sequence-specific information is lost at the digestion step.

### HPLC coupled to mass spectrometry

Coupling liquid chromatography with various types of mass-spectrometry (LC-MS and LC-MS/MS) is an extremely sensitive tool that can detect and quantify modified RNA nucleotides in the femto-to-attomole range. Initial steps of the protocol are performed as for the analysis of RNA nucleotide composition by LC/HPLC, which involves a total digestion of the RNA chain to mononucleotides and dephosphorylation to nucleosides. Separation of nucleosides using an RP column and coupling it to MS/MS analysis allows for a better characterization of the numerous oxidation products in DNA and RNA.^172–175^

In the MS/MS setup, the modified nucleosides can be analyzed using either the ‘dynamic multiple reaction monitoring’ (DMRM) or ‘neutral loss scan’ (NLS) techniques.^166^ DMRM is better suited for quantifying RNA modifications with known mass transitions upon fragmentation and known retention times. Multiple modified nucleosides with distinct retention time can be simultaneously analyzed in this mode. In contrast, NLS is effective for identifying novel non-canonical nucleosides with unknown mass transitions and retention times, but it is not effective for quantification.

The LC-MS/MS approach has been extensively validated across multiple laboratories for the detection of oxo^8^G and other advanced guanine lesions, such as Gh and Fapy-G, as well as adenosine oxidation products like oxo^8^A and Fapy-A in biologically relevant RNA samples. Furthermore, chlorinated nucleosides including Cl^8^G, Cl^8^A, and Cl^5^C have also been detected in RNA *via* LC-MS/MS.^69,116^

While LC-MS/MS efficiently identifies and quantifies oxidatively modified bases in RNA, it does not provide information about the specific transcript carrying the modified nucleoside or its exact location within the nucleic acid sequence. Further extending the LC-MS/MS-based sequencing approach could enable more precise mapping of guanine oxidation products. However, optimizing such analyses may be difficult because oxidized lesions are likely sub-stoichiometric and randomly distributed across the transcriptome compared to almost stoichiometric, localized enzymatic RNA modifications.

### RNA enrichment using antibodies against damaged RNA nucleotides

Immunochemical detection of biomolecules by specific antibodies is one of the major approaches in biochemistry and molecular biology. Antibodies are well known for highly specific recognition of epitopes in proteins, polysaccharides, nucleic acids and synthetic molecules. In addition, ELISA-like or immuno-precipitation-based techniques do not require sophisticated equipment and are rather straightforward. Thus, specific polyclonal and monoclonal antibodies have been developed for a number of small biological molecules, including non-canonical residues in DNA or RNA.

A general protocol for antibody preparation consists in conjugation of the periodate-oxidized nucleoside to neutral protein (BSA, ovalbumin or casein) and immunization of suitable laboratory animals. Using this method, polyclonal^176^ and monoclonal^177^ antibodies against the 8-oxo-purine derivatives of guanine as well as adenine, were prepared and characterized. These antibodies as well as phage-derived fragment antigen-binding regions (abbreviated as Fabs or Phabs) were shown to be suitable for competitive radioimmunoassays (RIA) for detection of free nucleosides, but showed limited specificity and weak recognition of oxidized residues in intact nucleic acid.^178^ Higher specificity was reported for polyclonal antibodies raised using the corresponding ribonucleosides. These were reported to recognize lesions in DNA, albeit with lower efficiency in DNA duplex.^179^ Availability of specific anti-8-oxoG antibodies for DNA and RNA opened the way for systematic studies of RNA damage in in stress^180^ and diseases, for instance in atherosclerosis,^181,182^ or in Alzheimer disease.^183–185^ Despite these successful applications, affinity and selectivity of nucleotide-specific antibodies is not outstanding,^186–188^ presumably due to the relatively small size of the hapten to be recognized and the presence of high copy numbers of competing structures, *e.g.* undamaged guanosines.

### Molecular principles for the detection of abasic RNA sites

rAP sites are rather commonly found, and the absence of a nucleobase causes their riboses to enter into an equilibrium between a ring-closed hemiacetal and an open chain featuring an aldehyde. Their detection can be achieved using the electrophilic reactivity of this aldehyde towards so-called aldehyde reactive probes (ARP) (Fig. 5), containing a characteristic nitrogen supernucleophile forming an oximether adduct. These molecules have been first developed for analysis of DNA abasic sites (AP),^189,190^ but extended to RNA analysis. ARP can be coupled to biotin for selective enrichment and quantification of the fragments exposing abasic site using ELISA-like assay, or to the fluorescent dye, allowing even more sensitive detection.^191^ Sensitivity of detection is very high, considering that detection of femtomolar amounts of DNA abasic sites was reported. Only DNA abasic sites and 5-formyluracil were shown to react with ARP, other common damaged compounds, like formamidopyrimidine and deoxyribosylformamide were not reactive.^190^ Fluorescently labeled ARP can be also used for specific labeling of DNA abasic sites followed by analysis by capillary electrophoresis with laser-induced fluorescent detector.^192^ In this setup, ARP labeling allows detection of as low as 1 abasic site/1 000 000 nucleotides, allowing detection as low as ∼20 attomoles.

The use of ARP was further extended to analysis of RNA oxidation. Sensitivity of detection was shown to be high (∼10 femtomoles as detection threshold) and ARP labeling was successfully used for monitoring of RNA damage upon exposure to Fenton reagent, γ-irradiation or peroxynitrite.^72^ ARP coupled to biotin was used for selective pull-down of RNA species containing abasic sites and RT-PCR analysis indicated enrichment of particular RNAs, allowing quantification of their oxidation level.^149^

ARP labeling of RNA abasic sites is a powerful method for global analysis of RNA oxidation, allowing also quantification of oxidation levels. However, in coupling to RT-PCR, the method is laborious and only oxidation status for a few RNA (mRNA) species can be assessed.

### Altered base-pairing properties of damaged and abasic RNA nucleotides as analytical principle

**Fig. 6:**
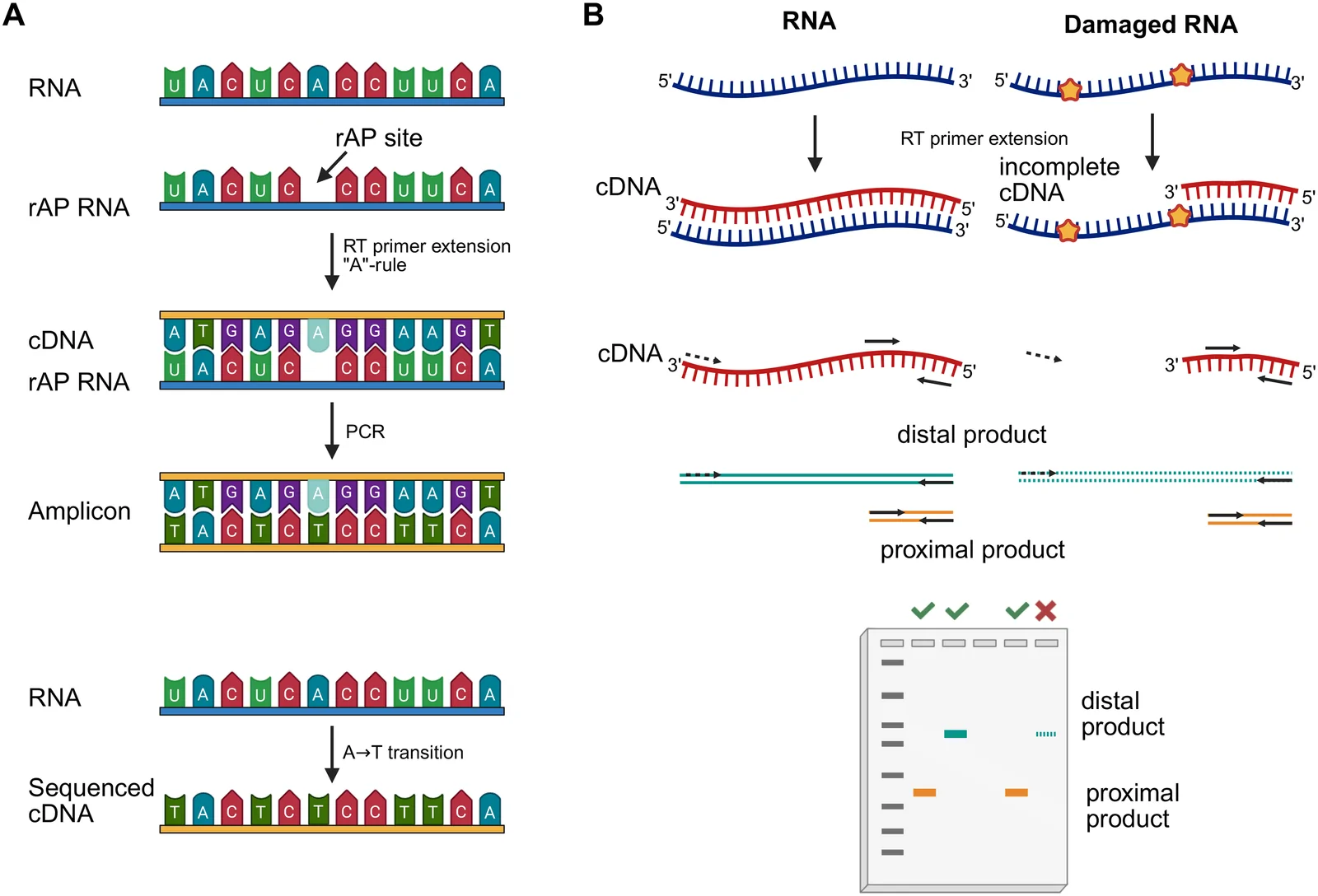
Analytical methods for RNA damage detection using RT primer extension. (A) Site-specific detection of rAP sites using the “A-rule”. Upon priming extension on rAP site-containing RNA, RT preferentially incorporates deoxyadenosine residue opposite to an rAP site in RNA template (this also applies to oxo^8^G). This creates an N → T transition in the sequencing of the resulting DNA. (B) PCR-based approach allowing detection and relative assessment of RNA damage level. Primer extension on damaged RNA stops at damaged nucleotides and thus is less efficient in producing full size cDNA products, compared to primer extension on undamaged RNA template. Longer PCR amplicons (distal product) are more affected and demonstrate the reduced yield, compared to short PCR products (proximal product). Analysis is generally performed using agarose or denaturing polyacrylamide gel electrophoresis. Created in BioRender. Weber, M. (2026) https://BioRender.com/8e82y6x.

Damaged nucleotides frequently show altered base-pairing properties, a feature that can be employed for their detection. Abasic DNA sites and damaged nucleotides are frequently by-passed by translesion DNA synthesis during replication, with the preferential incorporation of A nucleotide opposite to abasic site (so called “A-rule”)^193,194^(Fig. 6A). Since reverse transcriptases (RT) also insert “A” as a default nucleotide, formation of rAP sites, *e.g.* by rRNA 28S *N*-glycosylase activity of ricin, Shiga toxin and related ribosome-inactivating proteins can be also measured using primer extension by RT enzymes.^195^ PCR amplification methods with specific primers were developed to determine the proportion of cDNA with T → A mutations, *e.g.* RT-qPCR to assay *N*-glycosylase activity. Also, abasic sites present in DNA were shown to “base-pair” with bulky pyrene ring and the corresponding synthetic pyrene nucleoside triphosphate (dPTP) can be used by DNA polymerase which efficiently incorporates them into the daughter DNA strand opposite to DNA abasic site, albeit further extension is inhibited.^196^ This approach is appealing, but was not yet tested for rAP sites in RNA.

Another group of more general but less specific methods exploits globally reduced efficiency of reverse transcription upon cDNA synthesis from damaged RNA template. The by-pass of damaged RNA nucleotides by RT polymerase is only partial, and can thus be used for the estimation of the overall RNA damage^197^ (Fig. 6B). The presence of RT-arresting nucleotides, as well as RNA abasic sites and single-stranded breaks reduce synthesis of long cDNA molecules upon RT primer extension and this can be detected and measured as a ratio between proximal and distal PCR amplicons. This approach can provide some quantitative measure of damage; however, the method is non-specific for a given RNA damage product and the exact location(s) of damaged nucleotides cannot be determined precisely.

### Intercalation of fluorescent and spin-labeled probes into RNA:DNA duplexes

**Fig. 7:**
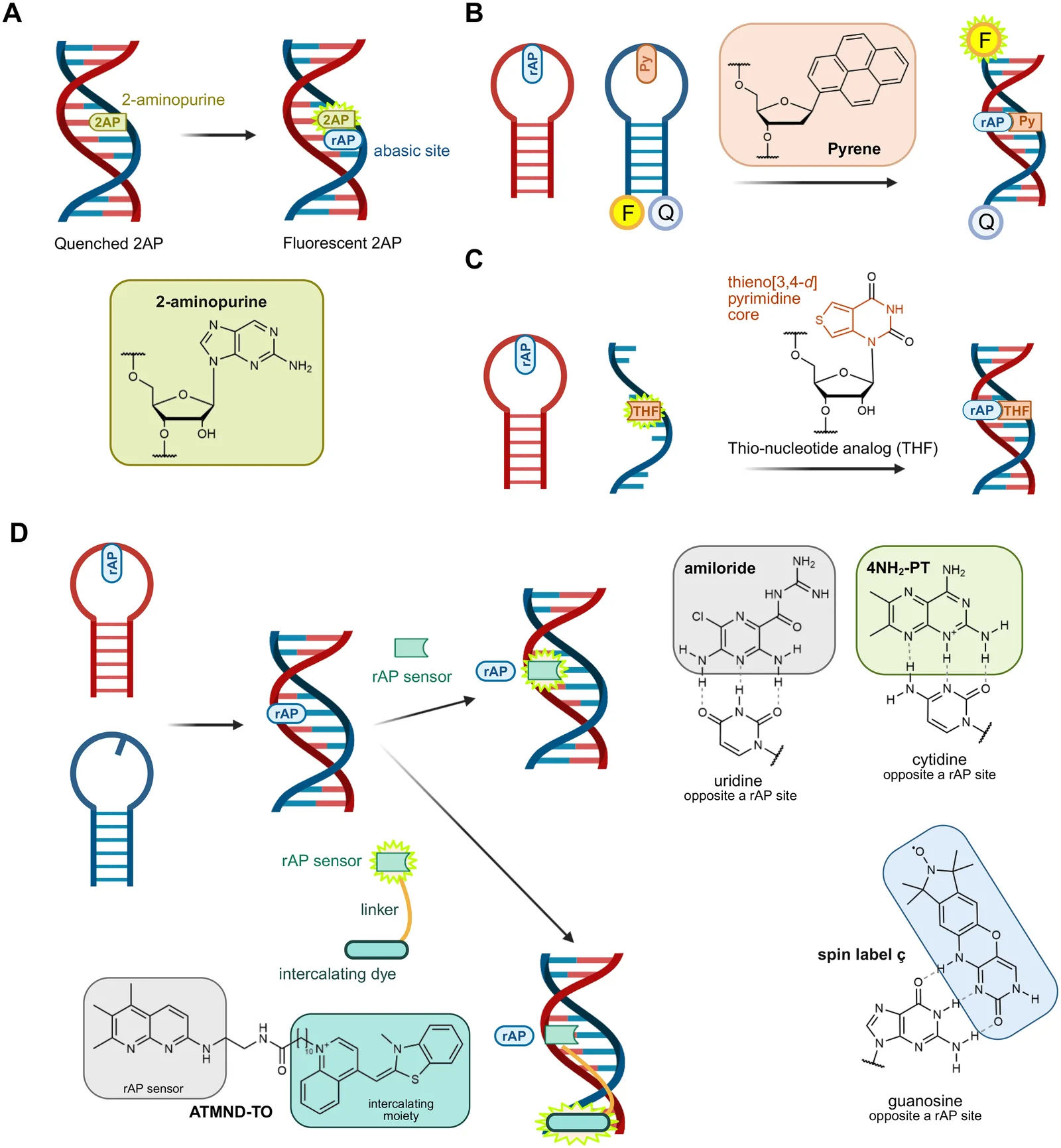
Specific detection of RNA abasic sites using intercalation and particular base-pairing with labeled nucleobases. (A) Detection of adjacent rAP site with 2-aminopurine (2AP) residue. 2AP is first incorporated into synthetic RNA by “RNA surgery” or direct chemical synthesis. When rAP site is generated side-by-side to 2AP, quenching of 2AP fluorescence is lost and such fluorescent signal can be detected. (B) RNA containing rAP site forms a duplex with complementary DNA oligonucleotide containing rAP probe, here pyrene (Py) residue, opposite to the rAP site. Negative control oligonucleotide contains normal base pairing nucleotide (not shown). Only pyrene (Py)-containing oligo forms a stable duplex with rAP RNA. Detection is based on fluorescent signal from fluorofore (F) which is not quenched by quencher (Q) in the duplex. (C) Fluorescent thio-nucleotide nucleotide analog (THF), is efficiently quenched when incorporated into perfect RNA:RNA or RNA:DNA duplex, but shows high fluorescence when it is opposite to RNA or DNA abasic site, allowing its detection and measurement. (D) rAP-containing RNA first forms a duplex with complementary DNA oligonucleotide. rAP site is detected by either baseparing of fluorescently (or spin) labeled nucleotide analog (amiloride, 4-NH2-PT, spin label ç), which is selected by complementarity to unpaired nucleotide opposing the rAP site (“orphan nucleotide”). Possible base pairing with U, C or G is shown. To improve the complex stability, rAP probe can be also linked to the duplex-intercalating moiety (bottom, 2-Amino-5,6,7-trimethyl-1,8-naphthyridine-thiazole orange conjugate, ATMND-TO), providing bi-valent interaction with the rAP-containing RNA. Created in BioRender. Weber, M. (2026) https://BioRender.com/4d3fokl.

Detection and quantification of RNA abasic sites and/or oxidized nucleotides can also be achieved by specific activation or quenching of fluorescent dyes upon incorporation into DNA:DNA or RNA:DNA duplexes. For instance, the fluorescence of 2-aminopurine (2AP) is enhanced when incorporated into DNA next to an abasic site (Fig. 7A). This effect can be used for kinetic studies on DNA repair enzymes.^198^

A number of proposed detection strategies are based on specific binding of labeled probes to the nucleotide (called also “orphan nucleotide”) facing the rAP site in the RNA duplex (Fig. 7B and C). In the simplest setup, the method uses nucleobase analogs capable of forming stable H-bonds with the nucleotide opposite to abasic site. In more sophisticated protocols, additional stabilization is provided by further intercalating agents or by formation of RNA/DNA duplexes bearing specific probes. Some of these methods have been only tested for DNA abasic sites, but one can reasonably expect similar behaviour for rAP sites as well.

An antisense approach for sequence-specific detection of an rAP with single-nucleotide resolution made use of the *N*-glycosylase activity of Ricin Toxin A-Chain in 28S rRNA^199^ to validate the application of DNA molecular beacons. The 14-mer DNA featured both fluorescent dye and its quencher at 5′- and 3′-ends, respectively (Fig. 7B). Such DNA beacon containing a dT at the probed position establishes a base-pair with the A nucleotide at the interrogated RNA target position and leads to a stable duplex, unquenching, and increase of fluorescence. In contrast, the same oligonucleotide does not form a stable interaction with an rAP at the target site and does not show fluorescence. A complementing DNA oligo with pyrene at the probing site behaves the opposite way, not basepairing with A in RNA but stably inserting into the abasic site, allowing discrimination by an increase of pyrene fluorescence. This method allows precise measurement of Ricin Toxin A-Chain activity, but requires specific design for every RNA abasic site to be measured or detected, and as such is not applicable in global screenings. Variations of such antisense approaches use nucleobase analogs with differential emission properties, such as a thio-nucleotide analog (based on thieno[3,4-*d*]pyrimidine) (Fig. 7C) incorporated into the complementary RNA. This fluorofore shows enhanced fluorescence when hybridized opposite an rAP site in an RNA: RNA duplex.^200^ In contrast, fluorescent 1-(2-deoxy-*b-d*-ribofuranosyl)-5-(benzofuran-2-yl)uracil undergoes quenching of fluorescence when placed opposite to rAP in a DNA: RNA duplex by an antisense DNA.^201,202^

Another conceptually similar approach uses the fluorescent 2-amino-5,6,7-trimethyl-1,8-naphthyridine (ATMND)^203^ which drastically quenches fluorescence upon pseudo base-pairing with C residue opposite rAPs in RNA: RNA duplexes.^204^ In the same vein, further compounds have been proposed, including nucleotide analogs amiloride or 2,4-diamino-6,7-dimethylpteridine (4-NH2-PT), which intercalate into the duplex instead of the missing base and base-pair with the orphan nucleotide in the opposite chain.^205,206^ EPR spectroscopy was also raised as a potential analytical method, based on the description of several spin-labelled compounds that can detect and identify local structural deformations in duplex DNA, including such caused by abasic sites, of which a compound termed ç is shown in Fig. 7D.^207–211^

Interaction of a molecular sensor for abasic site with the target RNA can be enhanced by conjugation with an intercalating dye *via* an appropriate linker. For instance, conjugation of ATMND or dimethylpterine (DMP) with cyanine dye (thiazole orange) resulted in nucleotide-specific probes, capable of distinguishing orphan nucleotides opposite to abasic sites.^212^ Linker variation can optimize the stability of the interaction, as shown for the “sensor” moiety ATMND and the intercalator thiazole orange^213^ (Fig. 7D bottom).

Interestingly, the strategy of specific complementarity was also implemented for detection of oxo^8^G in RNA, using a 2′-deoxyadenosine derivative with a diazaphenoxazine skeleton at the 6-amino group (Adap). oxo^8^G basepairs with the probe in *syn* conformation and a 2′-OMeRNA probe incorporating 2′-OMeAdap selectively detects damaged RNA residue in such heteroduplex.^214^

Overall, methods based on base-pairing and intercalation provide good selectivity and high sensitivity for rAP (and also oxo^8^G) detection. However, in addition to requiring substantial amounts of sample, synthetic DNA or RNA probes with incorporation of non-conventional nucleotides or analogs generally required custom syntheses, and these methods can only be used for previously defined and characterized rAP or damaged site.

### Modulation of the RNA nuclease cleavage by oxidized residues

Cleavage of modified/oxidized RNA by nucleases may be modulated by the presence of such damaged residues. For instance, RNAse T1 does not recognize sites containing oxo^8^G, while RNase A recognizes and cleaves RNA at such positions, albeit with modulated efficiency.^215^ Similarly, RNA exonuclease Xrn-1, a processive enzyme that catalyzes the hydrolysis of 5′-phosphorylated RNA in a 5′ → 3′ direction stalls (at least partially) at sites containing oxo^8^G. Yield of such stalled product with 5'-phosphorylated oxo^8^G varies, depending on the surrounding RNA sequence, hinting at specific detection.^216^ However, application of these observations as potential oxo^8^G detection methods was not further exploited.

### Mapping oxidatively modified residues in RNA by deep sequencing

With the advent of high-throughput sequencing (both second and third generation, NGS and NNGS, respectively), many of the classical detection methods described above, such as IP enrichment or RT signature-based approaches were adapted to NGS-based formats.

**Fig. 8:**
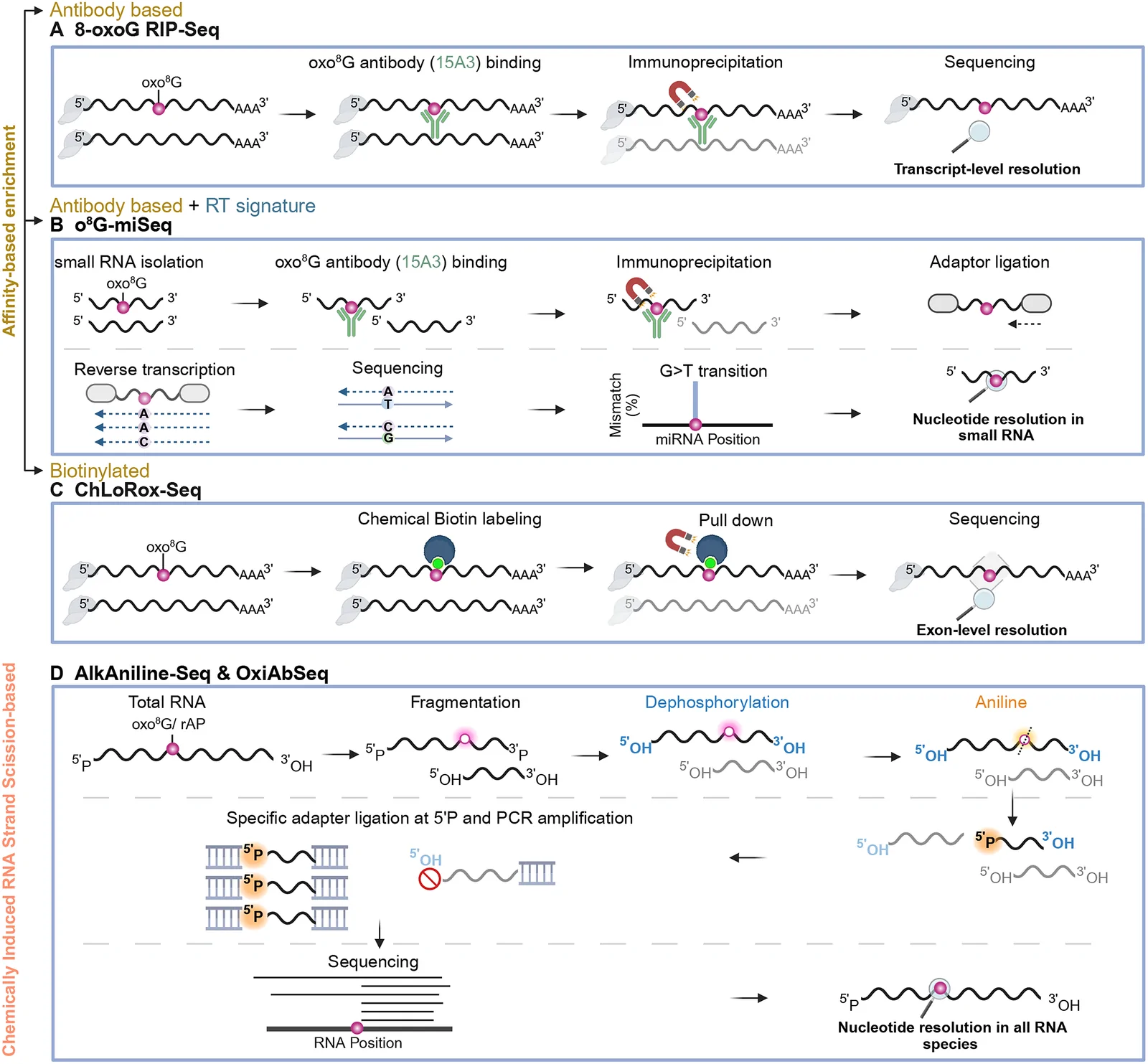
Current sequencing-based protocols for mapping oxidized RNA (mainly oxo^8^G and rAP sites). (A) RIP (RNA ImmunoPrecipitation) protocol with anti-oxo8G Ab (monoclonal 15A3 Antibody used here). As other RIP-like approaches, resolution is limited, generally to individual transcript or region, depending on fragmentation and library preparation protocol used. (B) RIP protocol with the same specific Ab (15A3) is coupled to analysis of RT-signature in cDNA after sequencing. Since oxo^8^G basepairs both with C and with A, G to T transitions are visible in the sequencing data and allow single nucleotide-resolution mapping of oxidized residues, applied here for miR. (C) In chemical-based ChLoRox-Seq protocol, oxo^8^G residues are first labeled by reactive primary amine bearing biotin. Enrichment is done by streptavidin-coated beads and enriched RNA is sequenced by standard NGS protocol. (D) AlkAnilineSeq and OxiAbSeq protocols for nucleotide-resolution mapping of rAP sites and oxo^8^G. After alkaline (in AlkAnilineSeq) or Mg^2+^ (in OxiAbSeq) fragmentation, RNA is de-phosphorylated and subjected to aniline cleavage, releasing 5′-P required for adapter ligation. AS the decisive molecular basis for high specificity, other fragments are not included in the library. While oxo^8^G produces only a moderate signal, rAP sites are detected with high efficiency. Created in BioRender. Raorane, K. (2026) https://BioRender.com/5u87am0.

MeRIP-like methods, employing polyclonal or monoclonal antibodies specific to a given RNA modification, were the first approaches coupled to short read sequencing. This way, immunoprecipitation of oxidized RNA with bead-immobilized anti-oxo^8^G antibody was used to characterize the transcriptome-wide profile of H_2_O_2_-driven oxidation of *S. cerevisiae* RNAs.^217^ The effect of a treatment of *S. cerevisiae* with H_2_O_2_ was reflected in an increase of the “RNA oxidation” index, a semi-quantitative parameter calculated as the ratio of normalized reads in the oxidized RNA to those in the total RNA. A similar MeRIP-based protocol was also used for analysis of RNA oxidation associated with exposure of lung cells to various pollutants and chemicals^67,218^ (Fig. 8A).

The weakness of antibody-driven enrichment protocols is related to lack of single nucleotide resolution in detection of the oxidized/damaged site in RNA. To gain such insight, enrichment with anti-oxo^8^G-Ab was coupled to analysis of RNA RT-signatures generated by oxo^8^G (G → T transitions, see above) in different miR species^219^ (Fig. 8B).

Ab-driven enrichment of modified RNAs frequently provide high background, due to small hapten size to be recognized and strong competition with unmodified residues in RNA. To improve the specificity of detection, chemical-based detection method, essentially derived from DNA-related protocol called “mapping of the oxidatively modified base 8-oxo-7,8-dihydroguanine (OG) by sequencing” (OG-Seq),^220^ was proposed also for oxo^8^G detection in RNA.^221^ In this ChLoRox-Seq protocol (for Chemical Labeling of RNA targeting 8-oxoG modifications and Sequencing), oxo^8^G residue is derivatized by biotinylated primary amine in the presence of one-electron oxidant potassium hexabromoiridate(iv) (K_2_IrBr_6_), allowing enrichment of oxidized RNA and analysis by NGS (Fig. 8C).

Another set of methods relies on specific RNA phosphodiester bond cleavage at oxidized sites upon exposure to chemical treatment or combination of chemical or enzymatic steps. Specific cleavage at abasic sites in RNA by aniline was already used in the late 70s, originally developed for direct chemical sequencing of RNA molecules.^222^ Indeed, treatment of RNA with combinations of alkylating and acylating species like DMS or DEPC on one hand, and nucleophilic species like hydrazine or sodium borohydride on the other hand, can be combined to either directly create abasic sites, or to convert methylated nucleotides to abasic sites. The latter applies *e.g.* to m^7^G which constitutes an RNA modification, but also represents a species resulting from metabolic alkylation. The above are susceptible to cleavage by aniline treatment. Conceptually based on this, an NGS approach used aniline cleavage after sodium borohydride (NaBH_4_) reduction. This allowed mapping of m^7^G based on rAP sites resulting from depurination of its reduction product.^223^ Since biotinylated ARP reacts with RNA abasic site, the reductive treatment with NaBH_4_ also allowed efficient pull down of m^7^G modified RNAs and all RNA molecules preexisting RNA abasic sites.^224^ Aniline cleavage was also used for NGS analysis of saporin (Ribosome-inactivating protein) cleavage specificity. Abasic RNA sites, created by *N*-glycosylase activity in rRNA, were mapped after aniline cleavage of de-purinated rRNA.^225^

With respect to guanosine oxidation, the Burrows’ lab investigated the efficiency of aniline-induced strand scission at different RNA oxidative lesions. Interestingly, while efficient cleavage was observed for high-level G oxidation products, oxo^8^G was resistant to aniline scission even upon prolonged (90 min) treatment.^100^

In our protocol called AlkAnilineSeq,^226^ aniline treatment is used in combination with mild alkaline hydrolysis, releasing certain naturally modified nucleobases to create rAP at sites of modifications including m^7^G, D, m^3^C and ho^5^C. It is noteworthy that ho^5^C is also found as a damaged/oxidized product in RNA (*vide supra*). Aniline treatment induces a cleavage that releases a downstream fragment containing a 5′-phosphate at the *N* + 1 nucleotide, providing molecular recognition for highly specific ligation of the sequencing adapter. Conversion to a library followed by sequencing allows very low background and highly specific detection of both oxidized RNA products and rAP at nucleotide resolution (Fig. 8D). We fortuitously discovered that AlkAnilineSeq efficiently detects tRNA rAP sites, generated by abortive TGT enzymatic reaction^227^ and build up a specific method for detection of oxo^8^G and rAP sites, protocol called OxiAbSeq (for Oxidized and Abasic sites Sequencing).^133^ In the OxiAbSeq protocol, alkaline fragmentation is replaced by Mg^2+^-driven fragmentation, reducing signals at naturally modified m^7^G and m^3^C. OxiAbSeq preferentially detects rAP sites, due to their high reactivity with aniline, while oxo^8^G residues give measurable, but much lower signals in sequencing. This protocol was successfully applied for analysis of rRNA, and also to miRNA and abundant mRNA species.

### Nanopore sequencing

Experimental evidence that DNA 8-oxo-dG as well as other dG-derived lesions in DNA can be detected by nanopore sequencing were provided by pioneering studies by the Burrows and White labs. Detection of 8-oxo-dG, and 5-guanidinohydantoin to iminoallantoin isomerization was thus studied using custom-made protein nanopore.^228–230^

Nowadays, commercialization of nanopore sequencing by Oxford Nanopore Technologies has made this extremely valuable tool available to a larger audience. The ensuing highly dynamic technology development produced direct sequencing of RNA, including a steadily growing number of non-canonical nucleotides. Current efforts focus mostly on RNA modifications including N6-methyladenosine (m^6^A), N7-methylguanosine (m^7^G), 5-methylcytosine (m^5^C), 5-hydroxymethylcytosine (hm^5^C), pseudouridine (ψ) and 2′-*O*-methylations.^231^ Given its use in the mapping of both natural modifications and oxidative damages (including 8-oxoG) in DNA^232^ a transposition to analysis of RNA oxidation is anticipated in future.^233,234^

## Biological consequences of RNA oxidation

RNA plays a pivotal role in gene regulation, translation and cellular signaling. RNA also serves as genetic material in certain viruses. Consequently, damage to RNA can contribute to numerous biological and pathophysiological outcomes. Oxidatively damaged RNA nucleosides have been observed in the context of ageing and various diseases including neurological diseases such as Alzheimer's disease (AD), Parkinson's disease, amyotrophic lateral sclerosis and dementia, atherosclerosis, cardiovascular disease, diabetes and various types of cancer.^59,180,183,235–246^ However, the exact mechanisms by which RNA damages are causative or consequence of diseases remain unclear. The emerging evidence suggesting that oxidatively damaged RNA species may impact cellular and molecular biological pathways is reviewed in this section.

### Altered basepairing and of damaged RNA

**Fig. 9:**
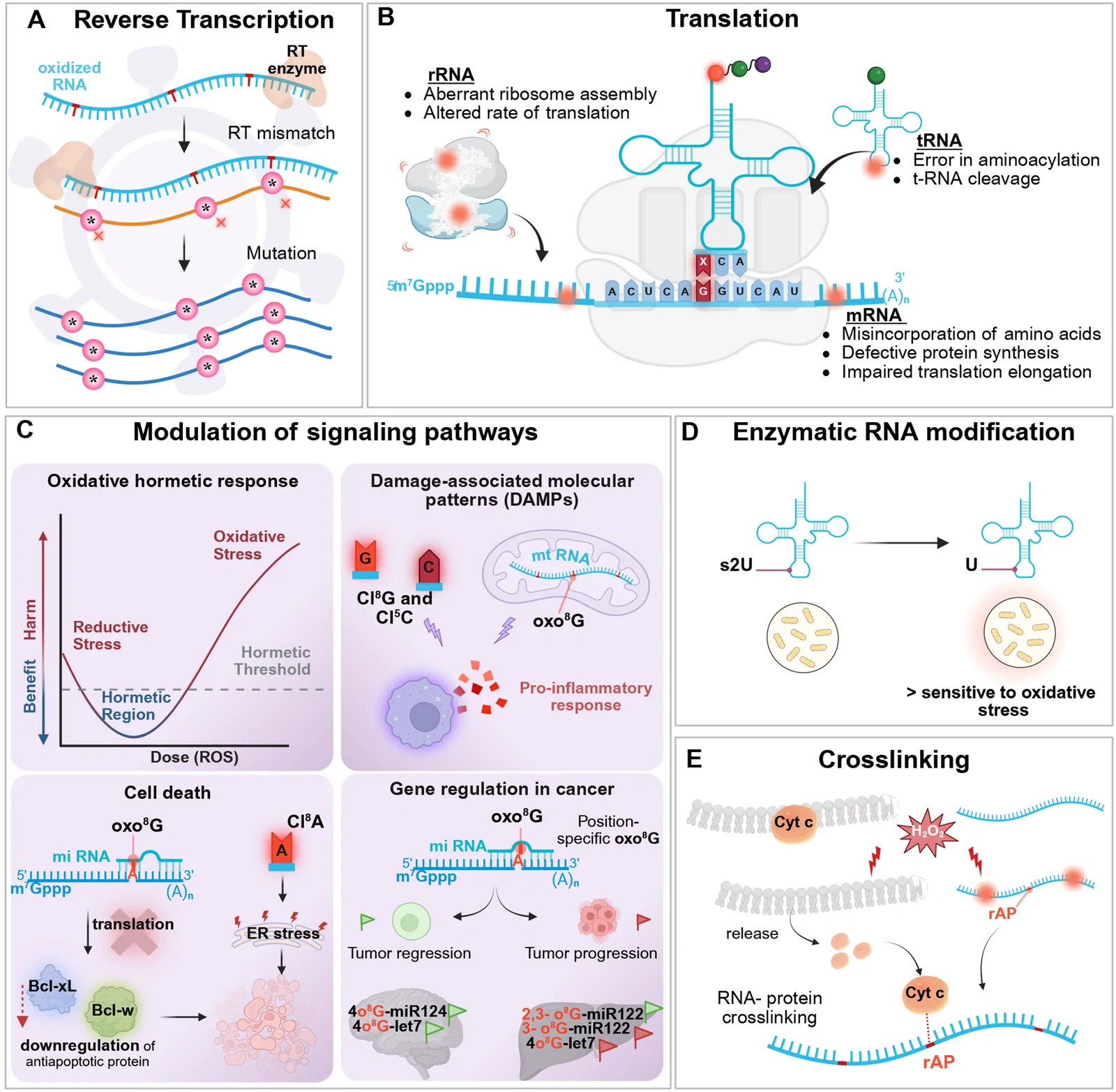
Biological impact of RNA oxidation. Oxidative damage to RNA disrupts critical molecular pathways that regulate gene expression and cellular homeostasis. (A) RNA oxidation induces mutagenesis during reverse transcription. Oxidative lesions such as oxo^8^G or rAP in genomic RNA can promote mispairing with A during reverse transcription, resulting in G to T misincorporation in cDNA. This mechanism may contribute to retroviral mutagenesis and accelerate mutation accumulation in retroviral genomes. (B) Translation fidelity compromised by oxidized RNA species. During translation, oxidative lesions in mRNA, tRNA, and rRNA can impair translational fidelity and efficiency, causing amino acid misincorporation, ribosome stalling, tRNA cleavage, and altered translation rates, ultimately leading to defective protein synthesis or global translational repression. (C) Oxidized RNA as modulators of cellular signaling pathways. Oxidized RNA and ribonucleotides can act as molecular signals that influence oxidative stress responses, immune regulation, and cell fate decisions. Top left: Oxidized rRNA generated under mild oxidative stress can trigger adaptive hormetic responses that promote cellular survival, whereas higher oxidative stress levels lead to cytotoxicity. Top right: Oxidized and chlorinated RNA and rNTPs can act as DAMPs, activating innate immune signaling pathways and pro-inflammatory responses. Bottom left: Oxidative modification of miRNAs can promote apoptosis through aberrant targeting and downregulation of anti-apoptotic proteins, while chlorinated ribonucleotides can induce apoptosis through disruption of cellular energy metabolism. Bottom right: Position-specific oxidation of miRNAs can alter target specificity and reprogram gene expression networks, contributing to either tumor progression or tumor regression. (D) Oxidation-induced loss of RNA modifications impairs cellular stress responses. Oxidative stress promotes desulfurization of modified tRNA nucleotides. Loss of enzymatic modifications compromises tRNA function and sensitizes cells to oxidative stress. (E) Oxidation-induced RNA–protein crosslinking. Oxidative generation of rAP sites promotes cytochrome *c*-tRNA crosslinking, potentially triggering apoptosis. Created in BioRender. Raorane, K. (2026) https://BioRender.com/c58uub6.

Many oxidized or otherwise damaged nucleotides feature altered hydrogen-bonding properties, and, as a consequence, altered basepairing, which may substantially differ from standard Watson–Crick patterns. Damage may be inflicted upon the existing RNA, or be generated by incorporation of damaged nucleotide triphosphates. Importantly, enzymes with surveillance functions have been described, which degrade the free oxidized nucleotides by hydrolyzing the phosphate group, thereby preventing the incorporation of such damaged nucleotides into DNA and RNA.^51,59^ Downstream effects arising of damaged RNA templates may include synthesis of inaccurate cRNA (*e.g.* by RNA polymerases from RNA viruses), or cDNA (by reverse transcriptases *e.g.* from retrotransposons, retroviruses,^247^ or telomerase activity). *In vitro* studies on HIV-1 RTase showed that it can readily incorporate dNTPs opposite to rAP.^248,249^ In retroviruses where RT occurs naturally as part of genome replication, the inherently low fidelity of viral RTases already contributes to high mutation rates. High promiscuity of lesion bypass on damaged templates suggests a plausible mechanism through which oxidative stress could accelerate retroviral evolution and promote the emergence of new variants^250^ (Fig. 9A). Both mechanisms pose significant challenges in antiviral therapy and vaccine development. On the other hand, pervasive oxidation of viral RNA constitutes an effective means of sterilization *e.g.* by HOCl or bleach^133,251,252^ and also happens *in vivo*.^116^

### Effect of RNA oxidation on translation

#### mRNA oxidation

Oxidized mRNA has been implicated in the pathogenesis of various diseases, including neurodegenerative diseases, cancer, cardiovascular diseases, and metabolic disorders.^253–255^ Detailed analyses of damaged biomolecules in AD brain and H_2_O_2_-treated primary cortical neuronal cells revealed a substantial amount of oxidatively damaged mRNA. Notably, certain mRNA transcripts, like those encoding antioxidant enzymes as SOD and GDH, appeared particularly susceptible to oxidative damage.^184,239^ While a direct connection between selective mRNA oxidation and pathological progression has been reported, the underlying mechanisms governing such preferential selection remain unknown.^255^

Various oxidative lesions have a distinct impact on translation. While certain base lesions such as oxo^8^G, oxo^8^A, ho^5^C and ho^5^U found in mRNA correlated with accumulation of truncated protein products during translation, the presence of rAP sites and etheno RNA lesions led to complete disruption of translation.^79^ Consistent observations demonstrated that oxo^8^G-containing mRNAs, when translated *in vitro*, produced reduced quantities of the encoded protein and resulted in protein aggregates, likely due to nascent protein chain misfolding. Similar results were reported *in vivo* when *in vitro* oxidized reporter mRNA was introduced into mammalian cells.^256^ Reduced protein production has also been shown in animals including the cuprizone-fed mouse model, postmortem MS brain and *in vitro* translation assay. Furthermore, accumulation of aberrant and misfolded proteins was reported in the cytoplasm, resulting in abnormal cytoplasmic foci formation (Fig. 9B).^257^ These effects may be driven by altered translation rates or translational errors arising from oxidized mRNA, however, the direct mechanistic link between RNA oxidation and processes leading to cytoplasmic foci formation remains poorly understood. At the molecular level, oxo^8^G in *syn*-conformation mispairs with A rather than C, potentially promoting altered decoding of mRNA by tRNA and consequently incorporation of “wrong” amino acids.^45,76^ However, under *in vitro* conditions, translation of oxo^8^G-containing mRNA stalled translation regardless of the relative position of oxo^8^G within the codon.^45^ Ribosome stalling was associated to a reduced peptide bond formation rate during translation elongation.^258^

In contrast, the relative position of rAP sites within the codon had a differential impact on translation in prokaryotic (bacterial) and eukaryotic (HEK293T) cell systems. While the presence of an rAP site (X̲) in the 3rd nucleotide position of the ‘AUX̲’ codon in the mRNA prevented translation in the eukaryotic system, the prokaryotic system was still able to translate, resulting in peptides with varying amino acids. This reflects an interesting difference in the tolerance of the two systems towards the presence of damaged sites in mRNA.^259^ The impact of rAP sites was also investigated on translation termination by inserting a triple ribose abasic sites (rAP–rAP–rAP) codon at the termination site.^260^ Despite observed ribosome stalling and the absence of aa-tRNA incorporation in the ribosomal A-site, both prokaryotic and eukaryotic release factors failed in their recognition of the abasic sites. Consequently, the nascent peptides remained trapped within the stalled ribosomes.^147,260^ Thus the presence of oxidatively damaged sites in mRNA can inhibit translation elongation and termination.^147^

In addition to oxo-derivatives, chlorinated nucleosides, such as Cl^5^C, have been identified by LC-MS/MS in the plasma of healthy donors.^152^ Such circulating chlorinated nucleosides can penetrate into the cell and be incorporated into DNA and RNA.^153^*In vitro* translation assays using reticulocyte lysate demonstrated that chlorinated mRNAs show 3.8-fold decrease compared to control unmodified RNA, indicating a notable impact on protein translation. However, the precise mechanism highlighting how the presence of chlorinated nucleotides in mRNA could impact translation remains to be fully elucidated.

#### tRNA oxidation

Oxidative stress is frequently associated with decreased tRNA abundance in prokaryotes^261–265^ and eukaryotes^266^ also in the context of disease such as AD.^244^ For both kingdoms, several reports suggest that the decline is not global but, instead, oxidative stress affects certain tRNA species stronger than others, and reportedly involves selective cleavage of certain tRNAs whose identity is conserved across different eukaryotic species (Fig. 9B).^265–267^

Oxidation experiments of tRNAs with H_2_O_2_*in vitro* resulted in comparable levels of oxo^8^G in both native and denatured tRNA, indicating at best moderate protection against oxidation by RNA structure. On the other hand, photooxidation produced more oxidative lesions in less structured tRNA regions like the anticodon and variable loops.^93^ Potential detrimental effects of such oxidative lesions include errors in decoding or aminoacylation, respectively (Fig. 9B).^44,52,265^

Of the known RNA enzymatic modifications, over >90 are exclusive to tRNA, playing essential roles in tRNA stability, folding and tRNA key functions in protein translation and beyond.^142,268^ Some are prone to damage upon spontaneous oxidation and thus potentially altered tRNA function.^142,143,266,269^ For instance, the oxidation product of 2-thiouridine, 4-pyrimidinone ribofuranoside (h^2^U) leads to altered base-pairing properties compared to its precursor, preferentially base pairing with G instead of A.^139^ This alteration of s^2^U derivatives at the anticodon nucleotide position 34 (wobble position) can result in miscoding.^59^ Oxidative desulphurization of s^4^U and s^2^U derivatives (mnm^5^s^2^U) is known to impact aminoacylation of certain *E. coli* tRNAs, particularly tRNA^Glu^, tRNA^Gln^ and tRNA^Lys^.^269^ Additionally, the absence of thio-group in 2-thiouridine (mnm^5^s^2^U) containing tRNA related to increased guanosine oxidation in *E. coli* under oxidative stress induced by UV radiation, and the lack of mnm^5^s^2^U modification in enzyme-deficient cell increased the sensitivity of *E. coli* to oxidative stress (Fig. 9D).^69^

### rRNA oxidation

rRNAs are highly structured and in the ribosome 3D structure, they are tightly bound to many ribosomal proteins. Such shielding from ROS in the cellular environment likely confers them increased stability compared to other cellular RNAs. In consequence, rRNAs isolated from *E.coli* cells under normal conditions were reported to be less damaged as compared to the non-ribosomal RNAs.^71^ However, a significant increase in rRNA oxidation levels was observed when isolated rRNA was subjected to H_2_O_2_ oxidation *in vitro*. This suggests that complex 2D and 3D structures may not necessarily protect rRNA during oxidative stress observed *in vitro*.^71^ Since rRNAs constitute the structural and functional core of the ribosome, damage to rRNA must have an obvious impact on protein translation.^44^ Oxidatively damaged rRNA nucleotides may also affect pre-rRNA maturation, leading to aberrant ribosome biogenesis, which in turn may affect translation (Fig. 9B). While there is an observed correlation between rRNA oxidation and abnormal ribosome biogenesis,^44,270,271^ direct evidence linking rRNA oxidation to impaired ribosome assembly is still lacking. Altered levels and damage to rRNAs, such as oxidised nucleosides or RNA chain cleavage, have also been observed in several age-related and neurodegenerative diseases.^245,272^ These alterations within the rRNAs are hypothesized to be causative of reduced or aberrant protein synthesis.

Although all rRNA species were found to be susceptible to oxidative damage, 23S rRNA, a component of the large subunit (LSU) in bacteria, is more prone to damage. Incorporation of oxo^8^G, oxo^8^A, ho^5^U, ho^5^C and abasic sites in *E. coli* 23S rRNA resulted in differential effects on translation, according to the location of incorporated modified nucleoside.^78^ Replacement of certain ribosome catalytic residues, such as A2451 and U2585, significantly affected protein synthesis at various stages of translation elongation. In contrast, other sites, like A2602, U2506, and G2447 were more tolerant and showed low or no effect upon inserting oxidative modifications. In particular, the presence of oxo^8^A at position 2451 did not impede tRNA binding or its translocation from the A site to the P site in the ribosome, but significantly affected transpeptidation, the process of peptide bond formations that relies on the 2′-OH group for proton relay. Notably, oxo^8^A is the first non-canonical nucleobase reported to affect this step. However, the presence of the abasic site at the same position (nt 2451) did not impact the transpeptidation activity. Analysis of SHAPE reactivity further suggested that oxidation at the C8 position of A2451 not only alters the electron distribution within the purine base, but could also affect the p*K*_a_ of its ribose 2′-OH group. In contrast, the presence of ho^5^U at position 2585 hindered tRNA accommodation at the A-site.^78^ These observations demonstrate that oxidative damage to rRNA may differentially affect the ribosome's properties, depending on the exact nature and location of the oxidative damage in the rRNA. Oxidation at some locations heavily impacts translation, while oxidative lesions at many other locations may be almost neutral. However, all these effects on translation were observed for *in vitro* study models and highlight the necessity of thorough investigation of their *in vivo* impact.

### Damaged ncRNAs in regulation of cellular signaling pathways

In addition to being involved in translation, numerous non-coding RNA species regulate cellular signaling pathways, thus the corresponding signaling networks may be impacted by oxidative RNA damage. Accumulating damaged RNA including rAP sites as well as oxidized and chlorinated nucleotides^273–275^ can trigger inflammatory immune responses or directly affect signalling. Circulating nucleic acids damage-associated molecular patterns (DAMPs) have been reported in the context of a wide range of inflammation-associated disorders, such as AD, atherosclerosis, *etc.* Depending on the oxidation level, typically measured by oxo^8^G accumulation, degraded and oxidized mitochondrial RNA (mtRNA) have been reported to modulate immune response by human and mouse macrophages^276^ (Fig. 9C). Various chlorinated nucleotides, which may result from HOCl oxidation are also known modulators of immune response. Exposure of murine macrophages to different chlorinated ribonucleosides had a pronounced effect on expression of pro-inflammatory cytokines and chemokines.^32^ In particular, a significant increase in the gene expression and protein levels for pro-IL-1β was observed after exposure of cells to Cl^8^G and Cl^5^C. The increase of IL-1β levels upon exposure to Cl^5^C was consistent with the nuclear translocation of transcriptional activator NF-κB rather than inflammasome activation. On the other hand, exposure of murine macrophages to Cl^8^G did not show increased nuclear translocation of NF-κB. This demonstrates the ability of specific chlorinated nucleosides to activate a pro-inflammatory signaling cascade that could contribute to the development of inflammatory pathologies such as atherosclerosis (Fig. 9C). Elevated levels of chlorinated nucleosides generated by RNA exposure to HOCl during atherosclerosis can also damage endothelial cells of arteries.^154,275^ A chlorinated derivative of A (Cl^8^A) was also shown to affect cell viability and metabolic activity,^277,278^*via* its conversion to Cl^8^ATP and reduction of ATP levels, thus affecting RNA synthesis and translation and inducing apoptosis (Fig. 9C).^154^

Oxidatively damaged miRNA may cause RNAi mediated erroneous degradation of mRNAs. Oxidation may cause altered basepairing of oxo^8^G or oxo^8^A and thus mistargeting in the binding of miRNA-containing RNA induced silencing complex (RISC) to mRNAs. This was reported for (miR184) promoting apoptosis by misrecognition and binding to mRNAs coding for anti-apoptotic proteins, Bcl-xL and Bcl-w (Fig. 9C).^279^ Similar mechanisms were reported to post-transcriptionally misregulate gene expression, leading to the development of diseases, including cancer.^219,236^ Examples concern miR124 and let7, as well as multiple sites in miR122^236^ (Fig. 9C). In mouse cardiomyocytes, specific oxidation of miR1 (7oxo^8^G-miR-1) is sufficient to drive cardiac hypertrophy.^219^

Oxidation of rRNA at low levels of oxidative stress in *S. cerevisiae* triggers specific cleavage of the 25S rRNA in the large subunit (LSU), at the evolutionarily conserved expansion segment 7 (ES7c) region. Such cleavage naturally occurs under respiratory growth conditions and does not impair translational competency or induce apoptosis. Instead, it induces an oxidative hormetic response, enabling cells to adapt and survive under conditions of severe oxidative stress^280^ (Fig. 9C).

While early investigations about the regulatory functions of oxidized RNA have largely been hypothesized based on observed correlations and co-occurrences, accumulated experimental evidence from oxo^8^G-modified miRNA studies underscores that position-specific oxidative lesions can directly modulate gene regulation, namely in cancer. A key future challenge in the RNA oxidation field is to uncover the precise roles and downstream signaling pathways associated with oxidatively damaged RNA and ribonucleotides, particularly in higher eukaryotes such as plants^273^ and mammals.

Oxidative stress was reported to induce crosslinks between biomolecules, including proteins and DNA. DNA–protein crosslinks lead to formation of double-stranded breaks, global genome instability, and to impacts on cellular functions that can ultimately contribute to pathologies including cancer, cardiovascular diseases and neurological disorders.^281,282^ Surprisingly, only a few studies have examined the context of oxidation-induced RNA–protein crosslinks. A high rate of RNA–protein crosslinks was observed in yeast ribosomes oxidized *in vivo*, with the crosslink interactions in rRNA primarily occurring at guanosine residues. However, the underlying mechanism of RNA–protein crosslink formation remains unclear.^283^ Consistent with this observation, another study, conducted *in vitro*, showed that crosslinks formed upon oxidation between cytochrome *c* (cyt *c*) and tRNA were preferentially observed at oxidized guanosines that are depurinated to form rAP site (Fig. 9E). Furthermore, the dissociation of cyt *c* associated with liposomes and its subsequent crosslinking with tRNA under oxidative conditions suggested a potential mechanism by which oxidation-induced RNA–protein (cyt *c*) crosslinks may induce apoptosis.^146^ Oxidants that are commonly used as disinfectants (like HOCl or ONOOH) can also promote crosslink formation between viral RNA and capsid proteins, resulting in inhibitions of viral RNA proliferation and inactivation of viruses.^251^

### Cellular mechanisms of coping with oxidized RNA

Unlike the numerous, redundant and sophisticated DNA repair pathways, no such dedicated repair mechanisms have been yet identified for oxidatively damaged RNA.^51,155,238^ Only some alkylated RNA residues (namely m^1^A, m^1^G and m^3^C) have been shown to be repaired by oxidative demethylation, catalyzed by AlkB protein homologs.^284–287^

Despite this, there are molecular mechanisms allowing specific recognition and elimination of oxidized/damaged RNA in the cell. First, apurinic/apyrimidinic endonuclease 1 (APE1), an enzyme known for cleaving DNA abasic sites, also recognizes rAP sites in RNA.^59,147,288^ This finding hints at a potential repair mechanism for oxidatively damaged RNA. Another surveillance mechanism reported to eliminate oxidized mRNA in yeast involves no-go decay (NGD): the ribosome-based mRNA quality control. Ribosome stalling, induced by translation of oxidized mRNA, triggers NGD proteins DOM34 and Cue2 that cleave and subsequently degrade the oxo^8^G-modified mRNA by Xrn1.^45,52^ In addition, certain RNA-binding proteins (RBP) selectively interact with oxidatively overmodified RNA and thus may help in recruiting or triggering decay or repair pathways (Table S1). For example, human AUF1 (also known as HNRNPD) recognizes oxo^8^G-containing RNA and participates in selective mRNA decay under oxidative conditions.^289^ Another example is Y-box binding protein-1 (YB-1) which binds to oxo^8^G-containing RNA. Under oxidative stress, the cells lacking YB-1 exhibited apoptosis^290^ suggesting a regulatory effect of such binding. Similarly, PCBP1 and PCBP2, two RBPs from the poly(C)-binding protein family bind to heavily oxidized RNA and modulate apoptosis under oxidative stress. Interestingly, these two proteins have opposing effects: while PCBP1 suppresses apoptosis, PCBP2 promotes it.^291,292^ In bacteria, polynucleotide phosphorylase (PNPase) is one of the most well-characterized proteins that recognizes oxo^8^G-modified RNA. PNPase directly interacts with DEAD box RNA helicase RhIB, which also shows high affinity to oxidized RNA, to degrade oxidized cellular RNA under oxidative stress.^293^ This selective binding and degradation of oxidized RNA might provide resistance to bacteria, especially extremophilic bacteria to cope with oxidative stress.

Oxidized RNA can be also generated during its biosynthesis by using already oxidized NTPs present in the nucleotide pool. MutT homologues have been identified in bacteria and humans that degrade the free oxidized nucleotides by hydrolyzing the phosphate group and thereby preventing the incorporation of such damaged nucleotides into DNA and RNA.^51,59^

## Conclusion

Spontaneous or chemical induced RNA modifications and universally present in all types or RNA species. Despite their physiological relevance and diverse implications in regulation of cell physiology, such damaged (in fact mostly oxidized) RNA nucleotides are unfairly underconsidered, compared to more studied, enzymatically formed, counterparts. Indeed, such spontaneous RNA damage, frequently related to cellular stress, remained largely understudied in the past decades. Elucidating various aspects of oxidative RNA damage, including chemistry and physiological importance of such nucleotides is necessary to understand their role in normal conditions, and numerous human pathologies. For a long time, progress in the field was hampered by the lack of appropriate methodology, allowing precise and specific mapping of RNA oxidation and related damage. The situation is rapidly changing now, with the emergence of high throughput mapping technologies, bases both of short-reads Illumina type sequencing and long-reads nanopore approach. Combining such powerful analytical methods will undoubtedly allow immense progress in the field and bring better understanding of the physiological role(s) and consequences of RNA oxidation.

## Conflicts of interest

Mark Helm is a consultant for Moderna Inc.

## Supplementary Material

CB-OLF-D6CB00143B-s001

## Data Availability

This review article does not use any unpublished data. Supplementary information (SI) is available. See DOI: https://doi.org/10.1039/d6cb00143b.
