# Supplementary material for: Chemistry and biology of oxidatively damaged RNA nucleotides
Source: RSC Chem Biol. 2026 Sep 1;7(10):1867–96. doi: 10.1039/d6cb00143b (PMC13555933; doi:10.1039/d6cb00143b)
Supplement: Supplementary file 1 [file CB-007-D6CB00143B-s001.pdf]

Supplementary Information (SI) for RSC Chemical Biology.
This journal is © The Royal Society of Chemistry 2026

Table S1: Proteins recognizing and binding to oxidized RNA

Protein (abrv)	Protein (full name)	Damaged nt (abrv)	Damaged nt (full name)	Biological system	Role	Ref
PCBP1	poly(C)-binding protein 1	oxo^{8}G	8- oxoguanosine	Human HeLa cells	Binds to heavily oxidized RNA. Induce apoptosis	(1, 2)
PCBP2	poly(C)-binding protein 2	oxo^{8}G	8- oxoguanosine	Human HeLa cells	Binds to heavily oxidized RNA. Suppress apoptosis	(1)
MutT	Nudix family protein	oxo^{8}G	8- oxoguanosine	*E. coli*	cleave ribonucleotides carrying 8-oxoG by hydrolyzing the oxidized nucleoside rNDPs or rNTPs to monophosphates preventing the incorporation of the free oxidized nucleotides into RNA synthesis.	(3–5)
AUF1	ribonucleoprotein complexes by AU-rich element RNA-binding protein 1	oxo^{8}G	8- oxoguanosine	Human HeLa cells	specifically binds to oxidized poly(A)-tailed mRNA exposed to low degrees of oxidative stress, in order to eliminate oxidatively damaged mRNA	(6)
PNPase	polynucleotide phosphorylase (exoribonuclease)	oxo^{8}G	8- oxoguanosine	*E. coli* and other bacteria such as *(Deinococcus radiodurans)*	reduce the cellular availability of oxo^{8}G-modified RNA under oxidative stress. essential to survival during oxidative stress. Interacts with RhlB and ribosomal protein S1(RpsA), forms a complex to process oxo^{8}G in RNA	(7)
APE1	apurinic/apyrimidinic endonuclease 1	rAP	abasic mRNA	Human HeLa cells	processes of rAP sites within mRNA transcripts	(4, 8, 9)
DOM34-Cue2 and Xrn1	proteins associted with NDG complex	oxo^{8}G	8- oxoguanosine	Yeast	recognizes stalled ribosomes and triggers the No-Go Decay (NGD) pathway , degrade the oxidized mRNA	(10–12)
RhlB	DEAD box RNA helicase	oxo^{8}G	8- oxoguanosine	*E. coli* and other bacteria such as *(Deinococcus radiodurans)*	reduce the cellular availability of 8-oxoG-modified RNA under oxidative stress. essential to survival during oxidative stress. interacts with PNPase and ribosomal protein S1(RpsA), forms a complex to process oxo8G in RNA	(7)
MutT1	MutT homolog 1	oxo^{8}G	8- oxoguanosine	Mammals	cleave ribonucleotides carrying 8-oxoG by hydrolyzing the oxidized nucleoside rNDPs or rNTPs to monophosphates preventing the incorporation of the free oxidized nucleotides into RNA synthesis.	(5)
YB-1	Y box-binding protein 1	oxo^{8}G	8- oxoguanosine	Mammals	binds specifically to oxo^{8}G and induces RNA decay or stabilization. supress apoptosis under oxidative stress.	(13)

REFERENCES:

1. Ishii,T., Igawa,T., Hayakawa,H., Fujita,T., Sekiguchi,M. and Nakabeppu,Y. (2020) PCBP1 and PCBP2 both bind heavily oxidized RNA but cause opposing outcomes, suppressing or increasing apoptosis under oxidative conditions. *J Biol Chem*, **295**, 12247–12261.

2. Ishii,T., Hayakawa,H., Igawa,T., Sekiguchi,T. and Sekiguchi,M. (2018) Specific binding of PCBP1 to heavily oxidized RNA to induce cell death. *Proc Natl Acad Sci U S A*, **115**, 6715–6720.

3. Hahm,J.Y., Park,J., Jang,E.-S. and Chi,S.W. (2022) 8-Oxoguanine: from oxidative damage to epigenetic and epitranscriptional modification. *Exp Mol Med*, **54**, 1626–1642.

4. Tanaka,M. and Chock,P.B. (2021) Oxidative Modifications of RNA and Its Potential Roles in Biosystem. *Front Mol Biosci*, **8**, 685331.

5. Sekiguchi,T., Ito,R., Hayakawa,H. and Sekiguchi,M. (2013) Elimination and utilization of oxidized guanine nucleotides in the synthesis of RNA and its precursors. *J Biol Chem*, **288**, 8128–8135.

6. Ishii,T., Hayakawa,H., Sekiguchi,T., Adachi,N. and Sekiguchi,M. (2015) Role of Auf1 in elimination of oxidatively damaged messenger RNA in human cells. *Free Radic Biol Med*, **79**, 109–116.

7. Han,R., Jiang,J., Fang,J. and Contreras,L.M. (2022) PNPase and RhlB Interact and Reduce the Cellular Availability of Oxidized RNA in Deinococcus radiodurans. *Microbiol Spectr*, **10**, e0214022.

8. Prashar,T., De La Selle,F. and Hudak,K.A. (2023) Abasic RNA: its formation and potential role in cellular stress response. *RNA Biol*, **20**, 348–358.

9. Liu,Y., Rodriguez,Y., Ross,R.L., Zhao,R., Watts,J.A., Grunseich,C., Bruzel,A., Li,D., Burdick,J.T., Prasad,R., *et al.* (2020) RNA abasic sites in yeast and human cells. *Proc Natl Acad Sci U S A*, **117**, 20689–20695.

10. Simms,C.L., Hudson,B.H., Mosior,J.W., Rangwala,A.S. and Zaher,H.S. (2014) An active role for the ribosome in determining the fate of oxidized mRNA. *Cell Rep*, **9**, 1256–1264.

11. Yan,L.L. and Zaher,H.S. (2019) How do cells cope with RNA damage and its consequences? *J Biol Chem*, **294**, 15158–15171.

12. Yan,L.L., Simms,C.L., McLoughlin,F., Vierstra,R.D. and Zaher,H.S. (2019) Oxidation and alkylation stresses activate ribosome-quality control. *Nat Commun*, **10**, 5611.

13. Hayakawa,H., Uchiumi,T., Fukuda,T., Ashizuka,M., Kohno,K., Kuwano,M. and Sekiguchi,M. (2002) Binding capacity of human YB-1 protein for RNA containing 8-oxoguanine. *Biochemistry*, **41**, 12739–12744.
